# A Gain-of-Function Mutation in *Tnni2* Impeded Bone Development through Increasing *Hif3a* Expression in DA2B Mice

**DOI:** 10.1371/journal.pgen.1004589

**Published:** 2014-10-23

**Authors:** Xiaoquan Zhu, Fengchao Wang, Yanyang Zhao, Peng Yang, Jun Chen, Hanzi Sun, Lei Liu, Wenjun Li, Lin Pan, Yanru Guo, Zhaohui Kou, Yu Zhang, Cheng Zhou, Jiang He, Xue Zhang, Jianxin Li, Weitian Han, Jian Li, Guanghui Liu, Shaorong Gao, Ze Yang

**Affiliations:** 1 Key Laboratory of Geriatrics, Beijing Hospital and Beijing Institute of Geriatrics, Ministry of Health, Beijing, China; 2 National Institute of Biological Sciences (NIBS), Beijing, China; 3 Clinical Institute of China-Japan Friendship Hospital, Beijing, China; 4 Department of Radiology, Beijing Hospital, Ministry of Health, Beijing, China; 5 Department of Epidemiology, School of Public Health and Tropical Medicine, Tulane University, New Orleans, Louisiana, United States of America; 6 Center for Genetic Medicine and State Key Laboratory of Medical Molecular Biology, Institute of Basic Medical Sciences, Chinese Academy of Medical Sciences & Peking Union Medical College, Beijing, China; 7 The Key Laboratory of Reproductive Health, Liaoning, China; 8 National Laboratory of Biomacromolecules, Institute of Biophysics, Chinese Academy of Sciences, Beijing, China; 9 School of Life Sciences and Technology, Tongji University, Shanghai, China; National Cancer Institute, United States of America

## Abstract

Distal arthrogryposis type 2B (DA2B) is an important genetic disorder in humans. However, the mechanisms governing this disease are not clearly understood. In this study, we generated knock-in mice carrying a DA2B mutation (K175del) in troponin I type 2 (skeletal, fast) (*TNNI2*), which encodes a fast-twitch skeletal muscle protein. *Tnni2^K175del^* mice (referred to as DA2B mice) showed typical DA2B phenotypes, including limb abnormality and small body size. However, the current knowledge concerning *TNNI2* could not explain the small body phenotype of DA2B mice. We found that *Tnni2* was expressed in the osteoblasts and chondrocytes of long bone growth plates. Expression profile analysis using radii and ulnae demonstrated that *Hif3a* expression was significantly increased in the *Tnni2^K175del^* mice. Chromatin immunoprecipitation assays indicated that both wild-type and mutant tnni2 protein can bind to the *Hif3a* promoter using mouse primary osteoblasts. Moreover, we showed that the mutant tnni2 protein had a higher capacity to transactivate *Hif3a* than the wild-type protein. The increased amount of hif3a resulted in impairment of angiogenesis, delay in endochondral ossification, and decrease in chondrocyte differentiation and osteoblast proliferation, suggesting that hif3a counteracted hif1a-induced *Vegf* expression in DA2B mice. Together, our data indicated that *Tnni2^K175del^* mutation led to abnormally increased hif3a and decreased vegf in bone, which explain, at least in part, the small body size of *Tnni2^K175del^* mice. Furthermore, our findings revealed a new function of tnni2 in the regulation of bone development, and the study of gain-of-function mutation in *Tnni2* in transgenic mice opens a new avenue to understand the pathological mechanism of human DA2B disorder.

## Author Summary

Distal arthrogryposis type 2B (DA2B) is an autosomal dominant genetic disorder. The typical clinical features of DA2B include hand and/or foot contracture and shortness of stature in patients. To date, mutations in *TNNI2* can explain approximately 20% of familial incidences of DA2B. *TNNI2* encodes a subunit of the Tn complex, which is required for calcium-dependent fast twitch muscle fiber contraction. In the absence of Ca^2+^ ions, TNNI2 impedes sarcomere contraction. Here, we reported a knock-in mouse carrying a DA2B mutation TNNI2 (K175del) had typical limb abnormality and small body size that observed in human DA2B. However, the small body did not seem to be convincingly explained using the present knowledge of TNNI2 associated skeletal muscle contraction. Our findings showed that the *Tnni2^K175del^* mutation impaired bone development of *Tnni2^K175del^* mice. Our data further showed that the mutant tnni2 protein had a higher capacity to transactivate *Hif3a* than the wild-type protein and led to a reduction in *Vegf* expression in bone of DA2B mice. Taken together, our findings demonstrated that the disease-associated *Tnni2^K175del^* mutation caused bone defects, which accounted for, at least in part, the small body size of *Tnni2^K175del^* mice. Our data also suggested, for the first time, a novel role of tnni2 in the regulation of bone development of mice by affecting *Hif-vegf* signaling.

## Introduction

Distal arthrogryposis type 2B (DA2B) (also called Sheldon-Hall syndrome, or SHS) is an autosomal dominant genetic disorder with marked genetic heterogeneity. The typical clinical phenotypes of DA2B patients include hand and/or foot contracture malformation and shortness of stature [1]–[3]. Approximately 20% of familial incidences of DA2B can be explained by mutations in *TNNI2* that encodes a subunit of the troponin complex consisting of troponin C, troponin I and troponin T. Troponin complex is set of muscle proteins that are part of the contractile apparatuses for contraction of fast skeletal muscle.

The previous knowledge shows that the two functional domains of TNNI2 are located at the N terminus (residues 96 to 115 and residues 140 to 148). In absence of Ca^2+^, TNNI2 prevents muscle contraction through binding to actin and tropomyosin using the two domains [4], [5]. However, to date, all of the etiological mutations reported in DA2B families [6]–[10] are mapped at the *TNNI2* C terminus that appears to regulate, in part, its sensitivity to Ca^2+^ ions [11], [12]. These *TNNI2* mutations include p.R156X, p.E167del, p.R174Q, p.K175del and p.K176del [6]–[10]. They are highly conserved in eukaryotes from zebrafish to humans. Evidence from *in vitro* cell assays has suggested that the p.R156X and p.R174Q mutations facilitate muscle contractility through increasing the sensitivity of TNNI2 to Ca^2+^ [13].

To investigate pathogenesis of DA2B, we generated genetically modified mice with an endogenous *Tnni2^K175del^* mutation, which had been mapped by our previous study, using targeted gene editing [10]. Besides limb deformity, we observed that both the homozygous and heterozygous mutant mice consistently showed a small body size that was similar to the reduced size phenotype in human DA2B. However, this phenotype did not seem to be convincingly explained using the present knowledge of TNNI2 associated skeletal muscle contraction. Thus, we hypothesized that an additional function of TNNI2 might be responsible for this abnormal body development. To test this possibility, we focused on investigating the potential mechanism that leads to the small body size phenotype in DA2B mice.

## Results

### DA2B mice showed a small body size phenotype

**Figure 1:**
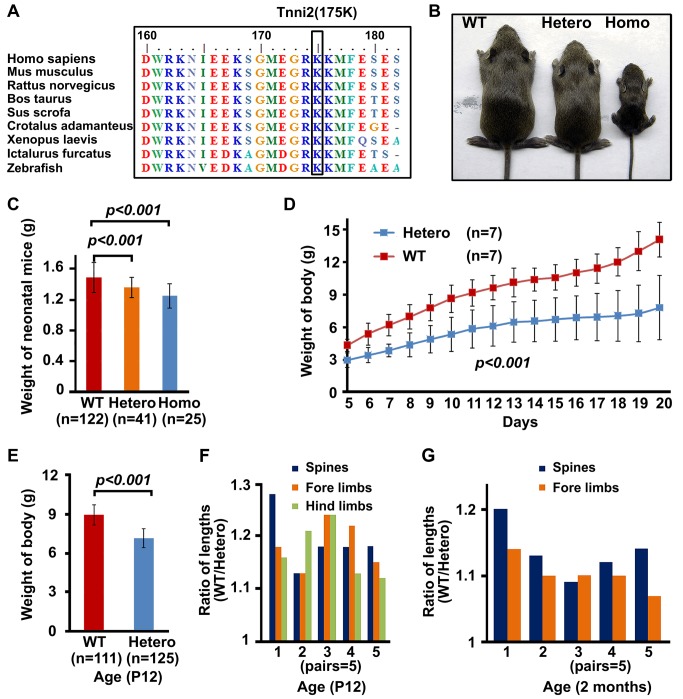
*Tnni2^K175del^* mutant mice showed small body size. (A) The conservation of the 175K amino acid in TNNI2 from humans to zebrafish. (B) P18 mutants exhibited smaller body sizes than wild-type littermates. (C) The comparison of weights of newborn wild-type and mutant mice. (D) The weight changes of heterozygous (n = 7) and wild-type mice littermates (n = 7) from P5 to P20. (E) Weights of heterozygous mutant mice (n = 125) and wild-type littermates (n = 111) at P12. (F) P12 heterozygous mice and wild-type littermates were sacrificed for measurement of the length of spines and limbs. Length ratio of the spines (blue), fore limbs (orange) and hind limbs (green) of wild-type mice (n = 5) to *Tnni2^K175del^* littermates (n = 5) at P12. (G) 2-month-old heterozygous mice and wild-type littermates were narcotized for measurement of the length of spines and limbs. Lengths ratio of the spines (blue) and fore limbs (orange) of heterozygous littermates (n = 5) to those of wild-type littermates (n = 5) at 2 months of age. Student *t*-test, mean±s.d. Postnatal day, P.

Using site-directed mutagenesis and targeted gene editing, we generated genetically modified mice with an endogenous *Tnni2^K175del^* mutation (DA2B mice) (Figure S1), which has been mapped to a large Chinese DA2B family in our previous study [10]. The 175K amino acid is highly conserved from zebrafish to humans (Figure 1A). Like DA2B patients, homozygous and heterozygous mutant mice exhibited an obviously smaller body size than their wild-type counterparts (Figure 1B and Figure S2B), as well as forelimb contracture (Figure S2A and C), supporting the idea that the mutation has a dominant effect on body development and limb formation. Newborn heterozygous (n = 41) and homozygous mice (n = 25) had lower birth weights (by approximately 10% and 15%, respectively) than wild-type littermates (n = 122) (*P*<0.001) (Figure 1C). The growth rates of heterozygous (*Tnni2^+/K175del^*) mice from post-neonatal day (P) 5 to P20 were significantly slower than wild-type controls (*P*<0.001, n = 7) (Figure 1D), and further analysis indicated that P12 *Tnni2^+/K175del^* mice (n = 125) were smaller and lighter than wild-type controls (n = 111) (*P*<0.001) (Figure 1E). The lengths of the spines, forelimbs and hindlimbs of P12 heterozygous mice were consistently shorter than those of their wild-type littermates (by 13–28% in the spines; by 13–24% in the forelimbs; and by 12–24% in the hindlimbs) (n = 5, Figure 1F). The length of the spine, fore limbs and hind limbs (Figure 1F) seemed to vary between the different pairs, and we considered that the difference between the different pairs of mutant mice could be due to individual variability in breastfeeding. However, the differences were not significant (for spines *P* = 0.21; for forelimbs *P* = 0.34; for hindlimbs *P* = 0.53). In addition, the respective lengths of the spines and forelimbs of *Tnni2^+/K175del^* mice were 9–14% and 7–14% shorter than wild-type littermates at 2 months (n = 5, Figure 1G). Except for the small body size and contracture in the forelimbs, we did not observe any limitations in movement of heterozygous or homozygous DA2B mice. Neither heterozygous nor homozygous embryos displayed differences in body size and weight relative to their wild-type littermates before embryonic day (E) 18.5 (Figure S3), indicating that mutant mice apparently experienced growth retardation during late embryonic development. Because the majority of homozygous *Tnni2^K175del/K175del^* mice always died within a few hours after birth and less than 2% of *Tnni2^K175del/K175del^* mice survived until weaning (Figure S4), subsequent studies were performed mainly using heterozygous mutants. Together, our data indicated that the phenotypes of *Tnni2^K175del^* mutant mice were similar to those in human.

### *Tnni2* was expressed in the osteoblasts and chondrocytes of the growth plate

**Figure 2:**
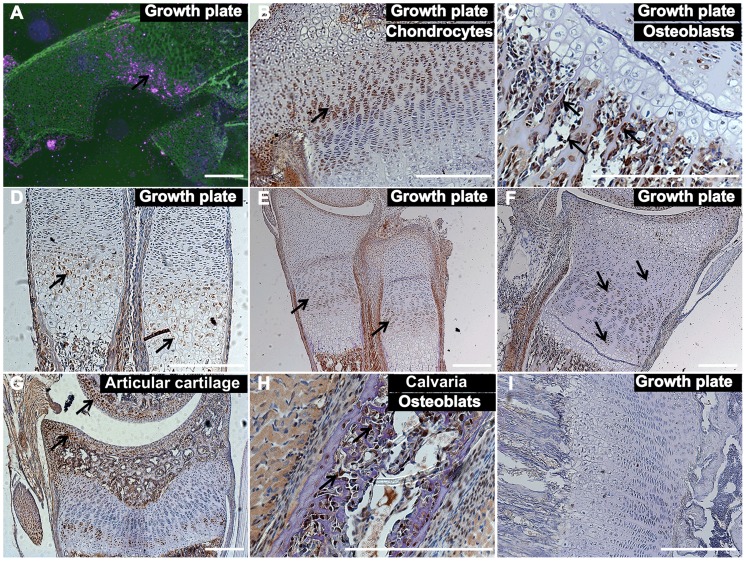
*Tnni2* was expressed in chondrocytes and osteoblasts. (A) In situ hybridization analyses with antisense *Tnni2* riboprobe on histological sections of wild-type radii of E15.5 embryos showed that *Tnni2* mRNA was expressed at the growth plates. Pink indicated *Tnni2* mRNA signal (*arrow*). (B–G) Immunostaining analyses of tnni2 expression in growth plate. (B–C) tnni2 was expressed at chondrocytes (B, *arrow*) and osteoblasts (C, *arrows*) of the tibia growth plates from P8 wild-type mice. (D–F) The expression of tnni2 in the growth plates of radii and ulnae exhibited a spatial and temporal change. (D) In E15.5 wild-type embryos, tnni2 was expressed in pre-hypertrophic and hypertrophic chondrocytes (*arrows*). (E) At E17.5 wild-type embryos, tnni2 was mainly observed in proliferating chondrocytes (*arrows*). (F) At P6 wild-type radii, expression of tnni2 was in pre-hypertrophic, hypertrophic, immature proliferating and rest chondrocytes (*arrows*), whereas no discernible tnni2 was observed in mature proliferative chondrocytes. (G) Expression of tnni2 was in articular chondrocytes of tibiae of P12 wild-type mice (*arrows*). (H) tnni2 was observed in calvarial osteoblasts of P6 wild-type mice (*arrows*). (I) Negative staining for tnni2 protein. Scale bar, 100 µm (A–I). Embryonic day, E.

Given that the known function of TNNI2 in regulating muscle contraction does not seem to reasonably explain the characteristic small body size that was observed in the DA2B mice and DA2B patients [14]–[15], we speculated that TNNI2 might have unknown roles in body development. Therefore, we initially performed *in situ* hybridization analyses to characterize the *Tnni2* expression in different organs from E15.5 wild-type embryos (Figure 2A, Figure S5). *Tnni2* mRNA was detected in the growth plates of long bones of radii and ulnae (Figure 2A), oesophagus (Figure S5B) and skeletal muscle as well (Figure S5G). Subsequent immunostaining analyses showed that tnni2 was present in the chondrocytes (Figure 2B) of growth plates and in the osteoblasts (Figure 2C) surrounding the border of the growth plates. Moreover, the expression of *Tnni2* in the growth plates exhibited a particular spatial and temporal change in wild-type mice (Figure 2D–F). In E15.5 wild-type embryos, tnni2 protein was observed in pre-hypertrophic and hypertrophic chondrocytes (Figure 2D), whereas at E17.5, tnni2 was mainly observed in proliferating cells zones (Figure 2E). After birth, tnni2 was expressed by resting, immature-proliferating, pre-hypertrophic and hypertrophic chondrocytes, but no discernible tnni2 was observed in mature proliferative chondrocytes (Figure 2F). In addition, we observed the expression of *Tnni2* in articular chondrocytes (Figure 2G) and calvarial osteoblasts (Figure 2H) of wild-type mice. The similar expression pattern was also observed in *Tnni2^K175del^* mice (Figure S6). Thus, the expression in the mouse growth plate and cranium suggested that the tnni2 protein might be involved in bone development.

### The bone development of DA2B mice was impeded

**Figure 3:**
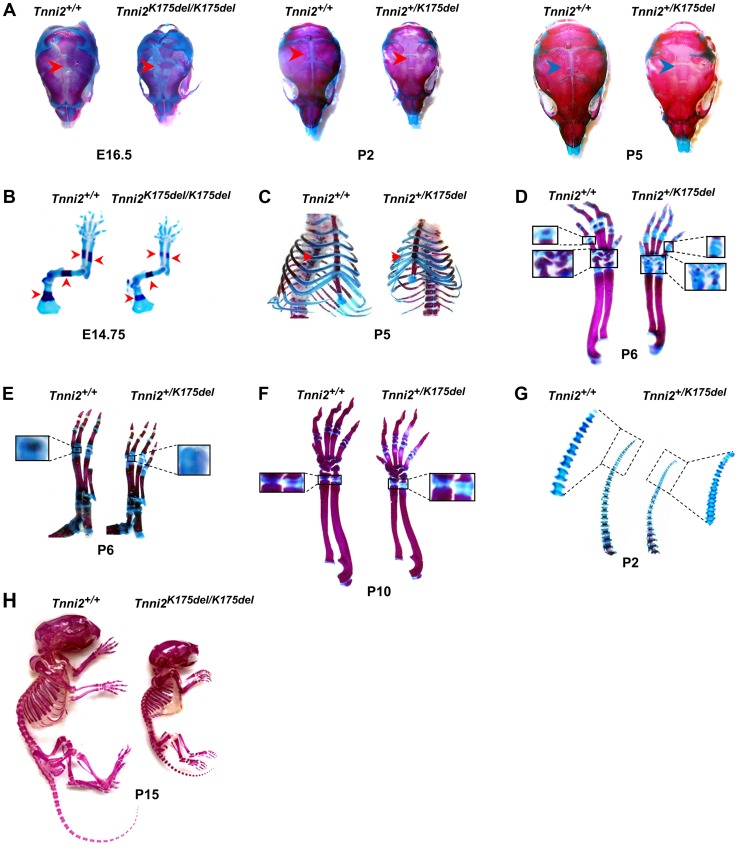
The *Tnni2^K175del^* mutation disturbed bone development of mice. Skeletons staining using Alcian blue for cartilage (blue) and Alizarin Red S for calcified bone (red). (A) Decreased mineralization (*arrows*) in the calvarias from *Tnni2^K175del/K175del^* mice at E16.5 and from *Tnni2^+K175del^* mice at P2 and P5 compared to their wild-type littermates, respectively. (B) The primary ossification centers (*arrows*) in forelimb bones from *Tnni2^K175del/K 175del^* mice at E14.75 were shorter than those of their wild-type controls. (C) Reduced ossification (*arrows*) in P5 mutant ribs. (D and E) At P6, the secondary ossification in phalanxes and carpals (D, the boxed regions) and metatarsal bones (E, the boxed regions) of *Tnni2^+/K175del^* mice were fewer than those of wild-type littermates. (F) Growth plates (the boxed regions) in radii and ulnae of heterozygous mutant mice at P10 were longer and less ossified than those of wild-type littermates. (G) Decrease in cartilage ossification in the P2 tails of mutants (the rectangle regions). (H) Skeletal preparation of homozygous mice and wild-type littermates at P15. All experiments were repeated using at least two pairs of samples.

We then investigated the bone morphology in DA2B mice by performing skeletal analyses. All skeletal elements of DA2B mice had a markedly smaller size and lower mineral content than those observed in wild-type mice. The degree of mineralization in the cranium was significantly reduced at E16.5 homozygous mutant embryos, P2 and P5 heterozygous mutant mice (Figure 3A and Figure S7), indicating that intramembranous ossification was delayed in *Tnni2^K175del^* mutants. At E14.75, the long bones of the forelimbs of *Tnni2^K175del/K175del^* embryos were smaller, and the primary centers of ossification in radii, ulnae, humeri and scapulae were evidently shorter than those in their wild-type littermates (Figure 3B and Figure S8). The heterozygous mutants had delayed cartilage ossifications in the ribs (Figure 3C), carpus and metacarpus (Figure 3D), phalanx (Figure 3E), growth plates of radii and ulnae (Figure 3F) and tail cartilage (Figure 3G) at P2, P5, P6, and P10. The primary and secondary ossification centers in the long bones of DA2B mice were impeded (Figure 3B–G), and their skeletal structures were smaller (Figure 3H), indicating that endochondral ossification was also impaired in *Tnni2^K175del^* mutants. Taken together, our data indicated that the *Tnni2^K175del^* mutation impeded the intramembranous (Figure 3A and Figure S7) and endochondral (Figure 3B–G) ossification of DA2B mice.

### The *Tnni2^K175del^* mutation disturbed the expression of molecules involved in *Hif*-signaling in the mutant bones

**Figure 4:**
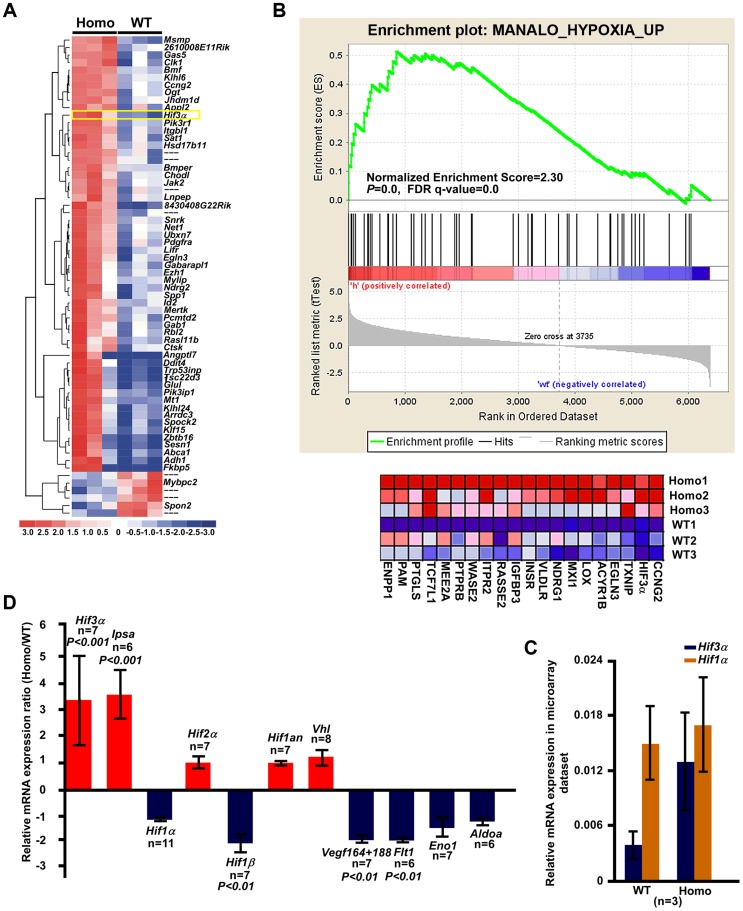
The *Hif3a* expression was significantly upregulated in the *Tnni2^K175del^* mutant long bones. (A) Heat map diagram of the 64 top ranking differentially expressed genes (Table S1) in 1-day-old homozygous mutant radii and ulnae (n = 3) compared to wild-type littermate controls (n = 3). Genes are shown in rows; samples are showed in columns. Red bar indicated higher expression and blue bar indicated lower expression. (B) GSEA plot (top) showed that differential expression of genes between the entire radii and ulnae from 1-day-old homozygous mice (n = 3) and wild-type littermates (n = 3) were significantly enriched in hypoxia induced gene set. Heat-map diagram (bottom) showed the 20 core-enriching genes in the leading edge. The number of permutation was 1000 times. (C) Relative mRNA expression of *Hif1a* and *Hif3a* (normalized to *Gapdh*) in our microarray dataset. (D) The relative mRNA expression of *Hif*-associated signaling molecules (normalized to *Gapdh*) in 1-day-old homozygous mutant radii and ulnae over those of wild-type littermates (Homo/WT), using qPCR analyses.

**Figure 5:**
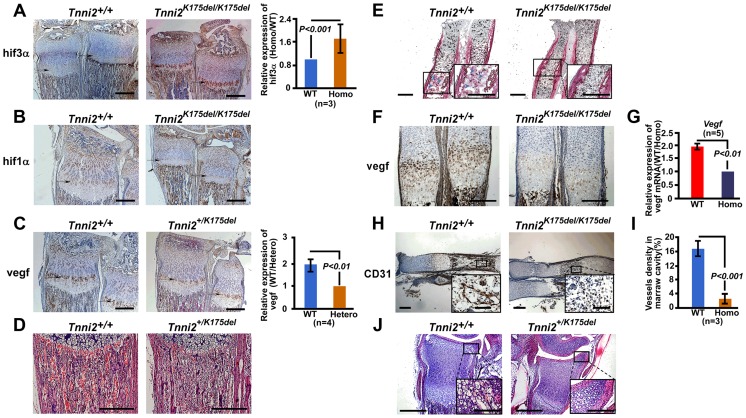
The disturbed *Hif* associated signaling in *Tnni2^K175del^* mice resulted in reduced angiogenesis and impeded formation of ossification centers of long bones. (A–B) Immunostaining analyses of the hif3a (A) and hif1a (B) expression in the hypertrophic cell zones (*arrows*) of radii and ulnae from *Tnni2^K175del/K175del^* mice and wild-type littermates at P12. *Right panel* of A: Quantification of hif3a expression (n = 3). (C) Immunostaining analyses revealed a decreased vegf in the hypertrophic cell zones of growth plates of radii and ulnae (*arrows*) from P12 heterozygous mutant mice compared to that of wild-type littermates. *Right panel* of C: Quantification of vegf expression (n = 3). (D) HE staining showed that the number of blood vessels surrounding trabecular bones were markedly decreased in radii from P12 mutants. Red blood cells were stained as red color. (E) Alkaline phosphatase staining exhibited a retarded formation of the primary ossification centers in radii of E15.5 homozygous mutants. Osteoblasts (blue *arrows*) have entered the marrow cavity and replaced the apoptotic chondrocytes in wild-type embryos but not in homozygous embryos. (F) Immunostaining revealed a decreased vegf in the hypertrophic cell zones of growth plates of radii and ulnae (*arrows*) from E15.5 homozygous mutant mice compared to those of wild-type littermates. (G) The relative mRNA expression of *Vegf* (normalized to *Gapdh*) in E15.5 homozygous mutant radii and ulnae over those of wild-type littermates (WT/Homo) using qPCR analyses. (H) CD31 stained the vessel endothelial cells in bone marrow of E15.5 homozygous mutant radii and wild-type controls. Insets showed magnified views of the box regions. (I) Quantification of blood vessel density in (H). (J) HE staining showed the growth plate of radii at P6. All experiments were repeated using at least three pairs of samples. Quantification analyses in A, C, G and I: Student's *t*-test, mean±s.d.; Scale bar, 100 µm (A–F, H, and J).

To characterize the molecules involved in the impeded bone development in *Tnni2^K175del^* mutant mice, we performed a microarray analysis using three pairs of intact radii and ulnas from 1-day-old *Tnni2^K175del/K175del^* and wild-type littermate mice. We tested differential gene expression using the SAM package. The *Tnni2^K175del^* mutation was associated with upregulation of 58 and downregulation of 6 probe sets (Score ≥3 and fold ≥2) (Figure 4A and Table S1). Furthermore, gene set enrichment analysis (GESA) of these mutant and wild-type samples showed significant enrichment of a set of hypoxia induced genes [16] (Normalized Enrichment Score = 2.30, *P* = 0.0, FDR q-value = 0.0) (Figure 4B). Hypoxia signaling, which is mainly mediated by hypoxia-inducible factor 1α (HIF1A) and its downstream target genes, including *VEGF* and its receptor *FLT1*, is important for bone development [17]–[20]. Notably, in our microarray dataset (SAM analysis, Table S1), *Hif3a* ranked in the top fifth of the significant differential expression genes. Of the genes that were observed to be dramatically upregulated in our microarray dataset, *Hif3a* had higher expression levels (more than 3-fold) in homozygous mutants than in the wild-type littermates (Figure 4C). HIF3A negatively regulates HIF1A signaling by binding to HIF1A or HIF1B and antagonizing HIF1-induced gene expression [21]–[22]. Therefore, we further examined the expression patterns of *Hif3a* and *Hif1a* in mRNA levels by qPCR in 1-day-old wild-type and mutant radii and ulnas (Figure 4D). Consistently, *Hif3a* and a main splicing variant of the *Hif3a* (*Ipas*) [23] were significantly increased by approximately 3.6-fold on average in homozygous mutant bones (Figure 4D). *Hif1a* expression was no significant difference between mutant bones and wild-type controls (Figure 4D). Furthermore, immunostaining showed that hif3a protein was significantly increased in the hypertrophic cell zones of mutant radii and ulnae compared to wild-type controls (Figure 5A). However, hif1a did not seem to differ in the growth plate of radii at P12 mutant and wild-type controls (Figure 5B). Thus, the marked increase in *Hif3a* expression in *Tnni2^K175del^* mutant bones suggested that *Hif*-signaling could be compromised in the mutant bones of DA2B mice.

We next tested the expression of molecules involved in *Hif*-signaling using qPCR analyses in 1-day-old homozygous mutant radii and ulnae and wild-type controls. The results showed that many hypoxia-associated genes, including *Hif1b*, *Vegf^164/188^*, and *Flt1*, were downregulated (approximately 1.85–2.2-fold) in homozygous mice compared to the wild-type controls (Figure 4D). Similarly, *Aldoa* and *Eno1*, two *Hif1a* target genes, were slightly decreased in *Tnni2^K175del^* mutants compared to controls (Figure 4D). In addition, the expression patterns of *Hif3a/Hif1a/Vegf/Flt1* in calvaria were consistent with those of the radii and ulnae (Figure S9). Taken together, our data suggested that the *Tnni2^K175del^* mutation disturbed the expression of molecules involved in *Hif*-signaling in the mutant bones.

*Tnni2^K175del^* mutation impeded the formation of primary and secondary ossification, and impaired chondrocyte differentiation and osteoblast proliferation in bones.

Vegf is important for bone development [18]–[20] and was significantly decreased in the bones of *Tnni2^K175del^* mice (Figure 4D and Figure S9). In accordance with diminished transcription, the vegf protein level was dramatically lower (96%, *P*<0.001, n = 4) at the hypertrophic cell zones of growth plates of radii and ulnae from heterozygous mutant mice than at those regions of wild-type mice (Figure 5C). As a consequence, fewer blood vessels were observed in the trabecular bones of heterozygous mutant mice at P12 than wild-type littermates (Figure 5D).

Vegf plays important roles in the formation of ossification centers [18], [24]. It is produced by hypertrophic chondrocytes, and then induces blood vessel invasion into the matrix of hypertrophic zones. In this study, we observed a delay in the formation of primary and secondary ossification centers in mutant mice (Figure 5E–J). At E15.5, apoptotic chondrocytes were replaced by osteoblasts in the cartilage of wild-type mice, but not in their *Tnni2^K175del/175K^* counterparts (Figure 5E). The vegf expression was reduced in growth plates of radii and ulnae from E15.5 homozygous mutant mice compared to that of wild-type littermates (Figure 5F and G). As a consequence, the recruitment of blood vessels into the cartilage plates was evidently fewer in E15.5 homozygous embryos than wild-type embryos (Figure 5H and I). At P6, early stage of the secondary ossification, the hypertrophy of growth plate chondrocytes and the invasion of vascular canals were normal in wild-type mice. However, these characteristics were not observed in heterozygous mutant mice (Figure 5J).

**Figure 6:**
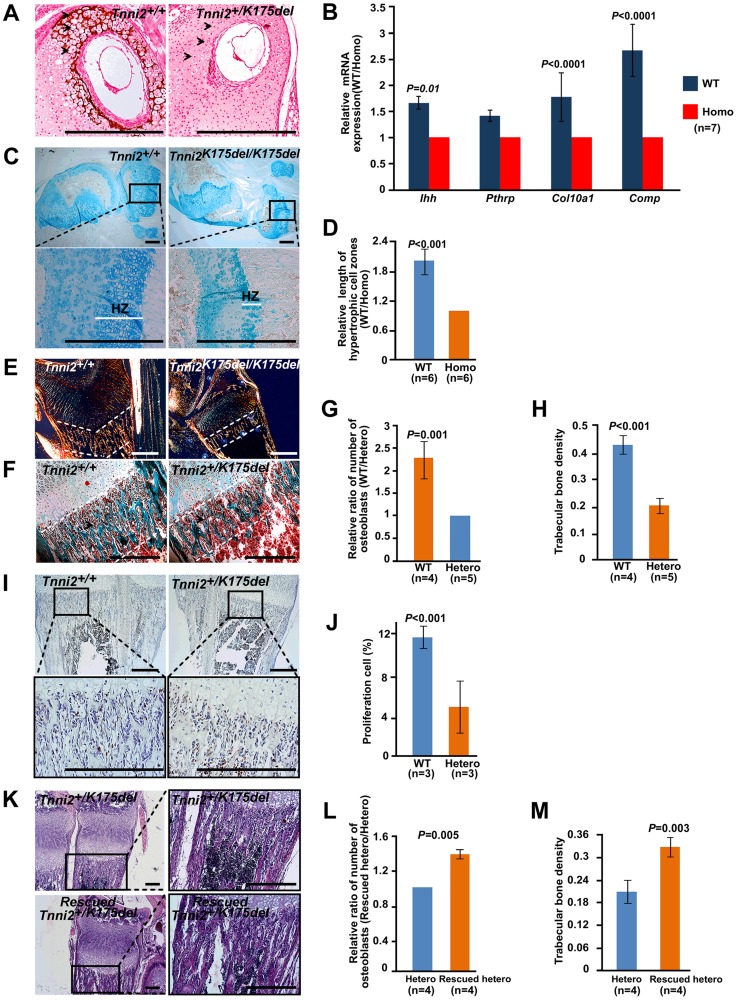
Chondrocytes differentiation and osteoblasts proliferation were impaired in mutant long bones. (A) Von Kossa stained at P6 nasal cartilage from *Tnni2^+/K175del^* mice and their wild-type littermates. Mineralization of the matrix and hypertrophic chondrocytes (*arrows*) were observed in the wild-type nasal cartilage, but few in mutant. (B) The relative mRNA expression of *Ihh*, *Pthrp*, *Col10a1* and *Comp* (normalized to *Gapdh*) in 1-day-old wild-type radii and ulnae over those of homozygous mutant littermates (WT/Homo), using qPCR analyses. (C) Alcian blue stained growth plate of tibiae. Hypertrophic zones (HZ) were markedly shorter in the growth plate of tibiae of *Tnni2^K175del/K175del^* mice at P12 than those of their wild-type littermates. (D) Quantification of the length of hypertrophic cell zones in (C) in growth plates of tibiae at p12 wild-type (n = 6) and *Tnni2^K175del/K175del^* mice (n = 6). (E) Polarization irradiation staining showed that type I collagen (orange) at trabecular bone domains (the regions between white dotted lines) in tibia of P12 homozygous mice was sparse relative to those of control littermates. (F) Masson's trichrome staining showed that the amount of osteoid and osteoblasts. At P6, the amount of osteoid was sparse (the regions between white dotted lines), and the number of osteoblasts (*arrows*) surrounding trabecular bone was decreased in radii of heterozygous mice compared to wild-type littermates. (G, H) Quantification of the number of osteoblasts surrounding trabecular bones (G) and the number of trabecular bones per unit area (H) at the radii and ulnae of *Tnni2^+/K175del^* mice (n = 5) and wild-type controls (n = 4) at P12. The trabecular bone and the number of osteoblasts were decreased on average by 53% and 57% in heterozygous DA2B mice, respectively. (I) BrdU incorporation to detect proliferating osteoblasts surrounding trabecular bones at P12. The number of osteoblasts in *Tnni2^+/K175del^* mice at P12 was significantly decreased relative to their wild-type littermates. (J) Quantification analysis of BrdU-positive cells in (I). The percentage of positive cells relative to total cell count was determined (n = 3). (K) HE staining showed that vegf treatment was able to partially rescue the number and thickness of trabecular bones and the number of osteoblasts in heterozygous mutants. (L, M) Quantification of the number of osteoblasts surrounding trabecular bones (L) and the number of trabecular bones per unit area (M) in (K) (n = 4). All experiments were repeated using at least three pairs of samples. B, D, G, H, J, L and M: Student's *t*-test, mean±s.d., Scale bar, 100 µm (A, C, E, F, I and K).

Vegf also promotes chondrocyte differentiation and stimulates osteoblast proliferation [24]–[26]. We next tested the effect of the decreased vegf on chondrocyte and osteoblast. We observed that the mineralization of the matrix and hypertrophic chondrocytes was absent at P6 nasal cartilage from *Tnni2^+/K175del^* mice compared to those of their wild-type littermates (Figure 6A). At 1-day-old homozygous mutant radii, the mineralization of the matrix at hypertrophic cells was fewer than that of wild-type littermate bones (Figure S10). Notably, the lengths of the hypertrophic cartilage zones in homozygous mice were significantly shorter than those of wild-type controls (Figure 6C and D). Furthermore, we tested the expression of differential chondrocyte markers using qPCR. The results showed that *Ihh*, *Pthrp*, *Col10a1* and *Comp* were decreased in radii and ulnae from 1-day-old homozygous mutant mice (Figure 6B). Together, these observations indicated a delay in chondrocyte differentiation in DA2B mice.

Osteoblasts produce osteoid, which is mainly composed of type I collagen and is mineralized to form trabecular bone and compact bone [27]–[28]. We found that the type I collagen, trabecular bones and the number of osteoblasts surrounding the trabecular bones were significantly decreased in *Tnni2^+/K175del^* mutant radii and ulnae compared to those of wild-type littermates (Figure 6E–H). The *in vitro* cultures of primary osteoblasts showed that the differentiation of osteoblasts of the mutants had no appreciable effect on alkaline phosphatase expression compared to that of control cells (Figure S11). However, BrdU incorporation experiments showed that the proliferation rate of the osteoblasts in heterozygous mutant mice was markedly lower (by 2.4-fold) than that of wild-type controls (Figure 6I and J). These results suggested that a deficiency in proliferative osteoblasts in DA2B mice might, at least in part, contribute to a reduction in trabecular bone.

Taken together, our data showed that *Tnni2^K175del^* mutant mice exhibited an impairment of angiogenesis, delay in endochondral ossification, and decrease in chondrocyte differentiation and osteoblast proliferation, which were similar to those bone defects observed in *Vegf*-deficient mice [18], [29]. Subsequently, we administered recombinant vegf to heterozygous mutants from P4 to P12. Notably, after supplementation with recombinant mouse vegf, the number of osteoblasts surrounding the trabecular bone was increased by approximately 30% on average (Figure 6K and L), and the number and density of trabecular bones in the treated mice were increased by approximately 36% (Figure 6K and M). These observations further suggested that the reduced vegf could contribute to defective bone development of the *Tnni2^K175del^* mice.

### The gain-of-function *Tnni2^K175del^* mutation resulted in the enhanced ability to transactivate *Hif3a* and the reduced vegf expression

**Figure 7:**
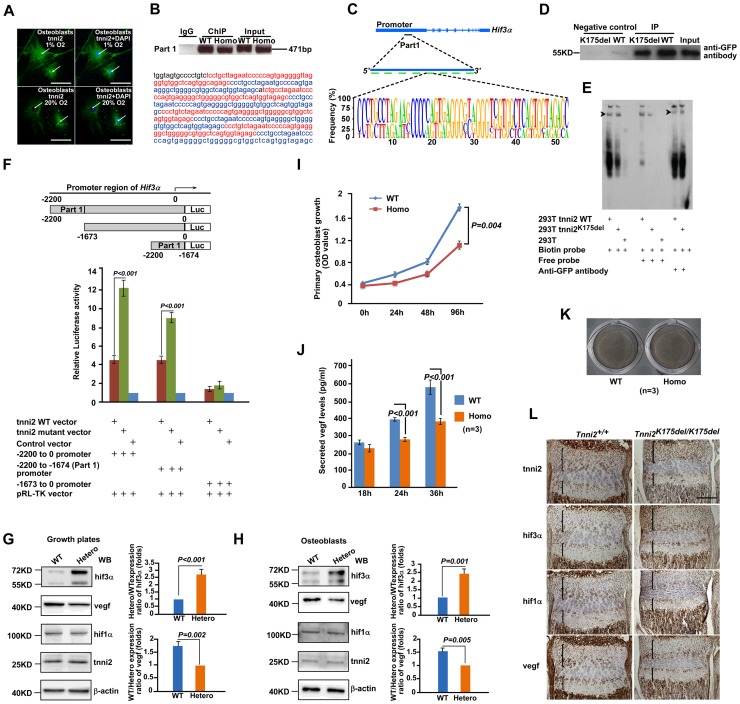
Mutant tnni2 protein increased hif3a and reduced vegf expression. (A) Immunofluorescence staining of tnni2 (*arrows*) in the cytoplasm and nucleus of the primary osteoblasts under both hypoxic (1% O_2_) and normoxic (20% O_2_) conditions. tnni2 was stained as green. Nuclei were stained with DAPI (blue). (B) *Upper panel*: Chromatin immunoprecipitation (ChIP)-PCR assay was performed using primary osteoblasts from 1-day-old *Tnni2^K175del/K175del^* mice and control littermates. Both wild-type tnni2 and tnni2^K175del^ mutant protein bond to the mouse *Hif3a* promoter by a 471 bp fragment (referred to as Part 1) *in vivo*; *Lower panel*: Nucleotide sequence of DNA of the Part 1. The eight highly conserved fragments were highlighted with Red or Blue color. (C) Schematic diagram of the Part 1. *Upper panel*: The position of Part 1 at *Hif3a* promoter. *Middle panel*: The Part 1 is consists of eight highly conserved fragments shown with green bars; *Lower panel*: The sequence feature of these repeat fragments. (D) DNA pull-down assays (DPDAs) performed with avidin-labeled dynabeads using the nuclear extracts (NE) from wild-type tnni2-GFP 293T stable cell line (WT) and NE from tnni2^K175del^-GFP 293 stable cell line (K175del). Biotin-labeled single repeat fragment of the Part 1 was used as DNA probe. Negative controls: wild-type or mutant NE incubated with non-labeled beads for nonspecific binding. Input: NE from wild-type TNNI2-GFP. Immunoblotting was performed using an anti-GFP antibody. (E) EMSA and Super-EMSA were performed using NE from wild-type tnni2-GFP 293T stable cell line and tnni2^K175del^-GFP 293T stable cell line. The DNA probe in (D) used here. (F) Luciferase assays of the truncated fragment (−1673 to 0 *Hif3a* promoter), the whole length fragment (−2200 to 0 *Hif3a* promoter) and the Part 1 fragment (−2200 to −1674 *Hif3a* promoter) were performed using 293T cell line under 20% O_2_ condition. Firefly Luciferase expression was measured and normalized by activity of Renilla Luciferase (pRP-TK). Ratio of the normalized Firefly Luciferase expression to that of control vector was used to represent relative luciferase activity. Data represents the mean±s.d. of five experiments carried out in quadruplicate. (G) Western blot analyses of tnni2, hif3a, vegf and hif1a in growth plates from P12 heterzyous mutant and wild-type littermate mice under normoxia condition. (H) Western blot analyses of tnni2, hif3a, vegf and hif1a in primary osteoblasts from newborn homozygous mutant and wild-type littermate mice. (I) MTT assay for testing proliferation of primary homozygous mutant osteoblasts and wild-type control (n = 3). (J) ELISA assay for testing secreted vegf in cell culture supernatant from primary homozygous mutant osteoblasts and wild-type control (n = 3). The secreted vegf levels of wild-type and mutant osteoblasts were normalized by cell number of the respective genotype at corresponding time point. (K) Von Kossa staining to test deposits of calcium of primary homozygous mutant osteoblasts and wild-type control. (L) Immunostaining for tnni2, hif3a, hif1a and vegf on consecutive sections of radii and ulnae of P12 homozygous mutant mice and wild-type littermate mice.

We next determined how the *Tnni2^K175del^* mutation resulted in an increase in *Hif3a* expression. A proteomic analysis was employed to identify novel TNNI2 interaction partners (Table S2). Immunoprecipitation followed by mass spectrometry analysis was performed using the nuclear extract from 293T cells ectopically expressing tnni2-GFP fusion protein. Interestingly, a group set of nucleocytoplasmic transporters including KPNB1, IPO5, IPO8, IPO9, TNPO1 and TNPO2, was identified as potential TNNI2 binding partner (Table S2), which suggested that tnni2 might be a nucleocytosolic shuttling protein and that its nuclear localization might be implicated in the regulation of *Hif3a* expression. To test this possibility, we examined the subcellular distribution of tnni2 in primary mouse osteoblasts. As shown in Figure 7A and Figure S12, tnni2 was observed in the cytoplasm and the nucleus of osteoblasts, and tnni2 nuclear distribution was independent of environmental oxygen tension.

Similar results were also observed in the primary *Tnni2^K175del^* mutant osteoblasts (Figure S13), and other cell types, such as 293T and HepG2 cells (Figure S14). To determine whether nuclear-localized tnni2 was involved in the transcriptional regulation of *Hif3a*, we performed chromatin immunoprecipitation (ChIP) using primary osteoblasts from the craniums of homozygous mutants and their wild-type littermates (Figure 7B and C, and Table S3). In Figure 7B, we identified a potential tnni2-binding fragment (471 bp, referred to as Part 1) residing in the *Hif3a* promoter region. The Part 1 consists of eight highly conserved sequences (Figure 7C). Our data suggested that wild-type and mutated tnni2 protein associate with the endogenous *Hif3a* promoter at similar strengths (Figure 7B). The binding of wild-type and mutated tnni2 to the *Hif3a* promoter was further verified by two *in vitro* experiments, DNA pull-down assays (DPDA) (Figure 7D) and electrophoretic mobility shift assays (EMSA) (Figure 7E). DPDA showed that the Part 1 was capable of pulling down both wild-type and mutant tnni2 from nuclear extracts of wild-type tnni2-GFP 293T stable line and tnni2^K175del^-GFP stable line. In addition, EMSA and Super-EMSA verified the physical interaction between tnni2 and the Part 1 fragment. Consistent with the ChIP results, the *in vitro* binding assays confirmed that wild-type and mutated tnni2 exhibited a comparable abilities to bind the *Hif3a* promoter. Furthermore, to test whether tnni2 was capable of activating the transcription of *Hif3a* through the Part 1, we constructed the whole length fragment (−2200 to 0 *Hif3a* promoter), the truncated fragment (−1673 to 0 *Hif3a* promoter) and Part 1 fragment (−2200 to −1674 *Hif3a* promoter) luciferase reporter vectors, and performed luciferase reporter assays. Our data showed that the whole length fragment and the Part 1 fragment both had significantly stronger luciferase activity compared with the truncated fragment (Figure 7F), and *Tnni2^K175del^* mutant exhibited a much stronger transactivation capacity (more than 2 folds) than its wild-type counterparts under normoxia (Figure 7F) and hypoxia (Figure S15). This finding indicated that the *Tnni2^K175del^* mutation enhanced the transactivation of tnni2 for *Hif3a* expression.

To further validate the effect of *Tnni2^k175del^* mutant protein on expression of *Hif3a*, *Hif1a* and *Vegf* in mutant bone, we performed western blot analyses using growth plates of radii and ulnae from P12 *Tnni2^+/K175del^* mutant mice and their wild-type littermates, primary osteoblasts from calvariae of newborn *Tnni2^K175del/K175del^* mutants and wild-type littermates, and primary chondrocytes from radii and ulnae of newborn *Tnni2^K175del/K175del^* mutants and wild-type littermate mice, respectively. The results consistently showed that the expression of hif3a was significantly increased, vegf was reduced and hif1a was not evidently altered in *Tnni2^K175del^* mutant growth plate and cells (Figure 7 G, H and Figure S16). Next, to show *in vivo* the potential interaction between tnni2, hif3a, hif1a and vegf, we performed a set of immunostaining on consecutive sections of radii and ulnae from P12 heterozygous mutant mice and wild-type controls. The staining displayed that the four proteins all were localized in resting, immature-proliferating, pre-hypertrophic and hypertrophic chondrocytes of the growth plates (Figure 7L). Moreover, to assess skeletal cell-specific role of the mutant tnni2, we conducted MTT and ELISA assays using primary osteoblasts harboring the *Tnni2^K175del/K175del^* mutant protein. Our data showed that the *Tnni2^K175del/K175del^* protein led to a reduction in proliferation and in secreted vegf levels of mutant osteoblasts (Figure 7I and J). Von Kossa staining displayed that no appreciable effect on mineral deposition in mutant cells compared to that of control cells (Figure 7K). Furthermore, we tested the expression of differential osteoblast markers using qPCR. The data showed that *Ocn*, *Spp1* and *Ibsp* were no significant difference between mutant and wild-type osteoblasts (Figure S17). These data suggested that the mutant tnni2 did not seem to affect differentiation of mutant osteoblasts. Taken together, our data indicated that the mutant tnni2 protein had a higher capacity to transactivate *Hif3a* than the wild-type protein, resulting in a reduction in vegf expression in mutant bone.

## Discussion

In this study, we presented genetic evidence that the *Tnni2^K175del^* mutation impaired bone development and disturbed hif-vegf signaling in DA2B mice. Hypoxia-inducible factors (HIFs) belong to a group of heterodimeric transcription factors consisting of HIF1A, HIF2 A, HIF3A and HIF1B HIF1A or HIF2A binds to HIF1B and induces the transcription of targeted genes, such as VEGF, under hypoxic conditions. Unlike HIF1A and HIF2A, HIF3A can negatively regulate HIF1-mediated gene expression by combination with HIF1A or HIF1B [21], [23]. In the human renal cell carcinoma cells and osteosarcoma cells, overexpressed HIF3A evidently inhibited HIF1-induced *VEGF* expression [30]–[31]. In mouse, ipas, a main isoform of hif3a, is induced under hypoxic condition [23]. Makino et al. reported that ectopic expression of *Ipas* in hepatoma cells reduced vegf production under condition of hypoxia and significantly decreased tumor vascular density. Moreover, obvious angiogenesis was observed in mouse corneas treated with IPAS antisense oligonucleotide [23]. The results strongly suggested that HIF3A suppressed angiogenesis through negative regulation of VEGF expression. In this study, we found that the growth plates of mice expressed *Hif3a*. And the hif3a was significantly elevated in *Tnni2^K175del^* mutant growth plates, primary osteoblasts and chondrocytes. However, the hif1a expression was not different between mutant groups and their wild-type littermate groups. Together, our results suggested that hif3a might inhibit hif1a-induced *Vegf* expression in the mutant growth plates and cells. The suggestion was further supported by the data that tnni2, hif3a, hif1a and vegf being localized in the same chondrocytes zones of the growth plates (Fig. 7L).

Our data indicated that tnni2 regulates *Hif3a* expression at transcriptional level. The *Tnni2^K175del^* mutant protein had a higher capacity to transactivate *Hif3a* expression than wild-type protein. Our results showed that both wild-type and *Tnni2^K175del^* mutant protein had a comparable capacity of attaching the Part 1 fragment (Figure 7C) locating at the promoter of mouse *Hif3a* gene.

HIF1A-VEGF signaling mediates angiogenesis in osteogenesis and regulates the differentiation of chondrocytes and the proliferation of osteoblasts [17], [18], [24]–[27], [32]. In HIF1A or VEGF deficient mice, abundant researches have reported the impairment of bone development including diminished angiogenesis, delayed primary ossification centers, decreased mineralized extracellular matrix in chondrocytes, reduced osteoblasts proliferation and reduced trabecular bone formation [17], [18], [24]–[27], [32]. In the current study, *Tnni2^K175del^* mice showed bone development deficiencies that were similar to mice lacking *Hif1*a or *Vegf*. These data strongly suggested that the impairment of bone development in the *Tnni2^K175del^* mutant mice was mediated by the reduced *Vegf* expression. This suggestion was supported by the partial recovery of trabecular bone density and the number of osteoblasts when the *Tnni2^K175del^* mutant mice were treated with recombinant mouse vegf (Figure 6 K, L and M). Taken together, our data suggested that the abnormal bone development associated with the *Tnni2^K175del^* mutation could be partly attributed to the disturbed *Hif-Vegf* signaling.

In addition, *Vegf* and its receptor *Flt1* are crucial for osteoclast activity [19], [26]. However, we did not observe an evident difference in the number or size of osteoclasts between E16.5 homozygous mutant radii and wild-type controls using the tartrate-resistant acid phosphatase (TRAP) activity test (Figure S18A). We also tested expression of *Trap* and *Mmp9*, two marker genes of activated osteoclasts, in the radii and ulnae of homozygous mutant mice and their wild-type littermates. However, their expression had not obvious change between mutant bones and wild-type controls (Figure S18B and C). Besides *HIF1A*, *CBFA1*, also known as *RUNX2*, is important for regulating *VEGF* expression in bone development [33]. However, our microarray and immunostaining analyses did not show significant difference in the *Runx2* expression in the radii and ulnae from 1-day-old and E16.5 homozygous mutants compared to their wild-type controls (Figure S19).

In the study, we described that tnni2 expressed in the chondrocytes and osteoblasts of long bone growth plate of mouse. Furthermore, we observed a decreased angiogenesis in the cartilage plate of *Tnni2^K175del^* bones. The observation was in the agreement with the report from Marsha, et al [34]. Their findings showed that TNNI2 was present in human cartilage and angiogenesis in corneas was inhibited through administration of TNNI2. Notably the extent of suppression of angiogenesis in corneas which was administrated with TNNI2 was similar to that of ipas in corneas [23], suggesting that TNNI2 inhibited angiogenesis in corneas might be mediated by HIF3A Interestingly, three independent reports have shown that TNNI2 acted as an inhibitor of angiogenesis to inhibit tumor growth and metastasis [35]–[37]. However, the molecular mechanism by which TNNI2 suppresses angiogenesis in tumors remains unclear to date. Our findings may provide a clue for the pathological process.

In this study, we focused on the mechanism causing the small body size phenotype of DA2B mice. Our findings showed that the *Tnni2^K175del^* mutation led to small body size in DA2B mice by impairing bone development. However, it needs to further investigate whether the small body size phenotype is also resulted from other abnormal tissue function. Besides *TNNI2*, the patients with mutations of *MYH3* also show the short stature phenotype [38]. Yet, the molecular mechanism is unknown. Recently a study reported that myosin directly interacted with runx2 in rat osteoblasts and that the expression of myosin was associated with osteoblast differentiation *in vitro* [39]. These data suggests that MYH3 was possibly involved in bone development as well.

In summary, our data indicated that the disease-associated *Tnni2^K175del^* mutation resulted in an abnormal increase in hif3a and a reduction in *Vegf* expression in bone. Furthermore, the decreased vegf impeded bone development and contributed to the small body size phenotype of DA2B mice. Our findings demonstrated, for the first time, a novel role of Tnni2 in the regulation of bone development in mice. These findings might provide an insight into the short stature in human DA2B disorder.

## Materials and Methods

### Ethics statement

All animal procedures were approved by the Institutional Animal Care and Use Committee of National Institute of Biological Sciences (NIBS) (Beijing, China).

### Generation of Tnni2^K175del^ knock-in mice

Mouse R1 genomic DNA was used as a DNA template to amplify the *Tnni2* gene. The mutation of c.523–525del-aag (K175del) in *Tnni2* was produced by overlapping-PCR. The 5′ homology arm (long arm) consists of a 2-kb promoter region and the entire *Tnni2* genomic DNA sequence. The 3′ homology arm (short arm) begins 400 bp downstream of the *Tnni2*. 3′ UTR and extends to 2-kb-downstream sequence. The ploxp vector was used as the targeting vector. An *EcoR* V site was added to the reverse primer for the short arm of recombination to facilitate Southern blot analysis. The targeting construct was introduced into 129Sv embryonic stem cells by electroporation. Positive ESC lines were selected following G418 screening and were confirmed by PCR-sequencing. Correctly targeted clones were injected into blastocysts of C57BL/6 mice and transferred into uteri of pseudopregnant females. Chimeric males were mated with ICR mice, and PCR-sequencing and Southern blot were used to determine germ line transmission of the targeted allele. cDNA from the muscle tissue of *Tnni2^K175del/K175del^* mice was sequenced to validate the *Tnni2* transcript with the del-aag mutation.

### In situ hybridization

Expression of *Tnni2* in the brains, hearts, lungs, stomachs, spleens, skeletal muscles, kidneys, radii, and ulnae at E15.5 was detected using in situ hybridization as previously described [40]. A Tnni2 cDNA fragment of 546 bp was subcloned into pBluescript. Sense and antisense ^35^S-labeled RNA probes were generated by T7 and SP6 polymerases, respectively. The activity of the probes was approximately 2×10^9^ disintegrations/minute/µg. Frozen sections (10 µm) of organs of wild-type mice at E15.5 were mounted onto poly-L-lysine-coated slides and fixed in 4% paraformaldehyde diluted in PBS at 4°C for 15 minutes. After hybridization and washing, the slides were incubated with RNase A (20 µg/mL) at 37°C for 15 minutes. The hybrids with RNase A resistance were detected after 1 week of autoradiography using Kodak NTB-2 liquid emulsion. The slides were post-stained with hematoxylin and eosin.

### Skeletal preparation

To observe the development of the whole skeleton, we consecutively stained skeletons of wild-type and mutant mice from E14.5 to E18.5 and from P0 to P20 for 24 hours with Alcian blue (pH 5). The skeletons were fixed for 6 hours in 95% ethanol followed by staining with Alizarin Red S. The clarification of soft tissue was performed using 1% potassium hydroxide. Each bone staining experiment was performed at least using two pair of samples.

### Histological and immunohistochemical examinations

For histological examination, the tibiae, radii, ulnae, and skulls from mutant mice and wild-type littermates were fixed in 4% paraformaldehyde. Paraffin sections and ice sections (4 µm thickness) were stained with HE for histological structures. Von Kossa method was used to stain mineralized trabecular bones and cartilage matrix. Masson's trichrome and Picrosirius-polarization staining was performed to test type-I collagen at osteoid at P6, P7, and P12 mice. Alkaline phosphatase staining showed osteoblasts in E15.5 mice (Alkaline Phosphatase kit, Sigma-Aldrich, USA). Alcian blue was used to stain chondrocytes at P12, P13, and P19 mice. Immunohistochemical analyses were conducted using anti-runx2 (1/50, Santa Cruz Biotechnology, USA), anti-CD31 (1/100, Santa Cruz Biotechnology, USA), anti-hif3a (1/50, Abcam Biotech, Cambridge, UK), anti-hif1a (1/50, Abcam Biotech, Cambridge, UK), anti-vegf (1/50, Abcam Biotech, Cambridge, UK), anti-tnni2 (1/40, Merck Chemicals, Nottingham, UK) at E15.5 to E18.5, P0, P6 to P8, and P12 mutant mice and wild-type littermates. Whole radii and ulnae from E16.5 homozygous mutant and wild-type littermates were stained for TRAP activity using Sigma-Acid Phosphatase and Tartrate Resistant Acid Phosphatase Kit. TRAP-positive cells on bone surfaces that contained more than two nuclei were counted as osteoclasts. These histological and immunohistochemical analyses were performed using at least three biological repeats.

### Primary osteoblast culture

Osteoblasts were obtained from calvariae of newborn *Tnni2^K175del/K175del^* mutants and wild-type littermates. The experiment was performed as previously described [41]. Briefly, calvariae from newborn homozygous mutants and their wild-type littermates were carefully removed. They were digested for 2 hours at 37°C in DMEM containing 0.2% collagenase type I. Cells were isolated and cultured in DMEM containing 10% FBS. The primary osteoblasts from neonatal mice were inoculated at a density of 10^5^ cells per dish in 6-cm dishes. The primary osteoblastic cells were placed at a density of 5×10^4^ cells per well in a 24-well plate and cultured for 48 hours and ALP activity were assessed using the Sigma-Aldrich Alkaline Phosphatase kit. The primary wild-type and homozygous mutant osteoblasts were cultured in the presence of 10 mM β-glycerophosphate and 50 µg/mL ascorbic acid to 14 days, and then were stained using Von Kossa method to test minerization and differential osteoblast markers.

### Immunofluorescent staining

Primary osteoblasts were grown on cover glasses under 1% O_2_ or 20% O_2_ conditions for 30 hours. The cells were fixed with 4% paraformaldehyde for 2 hours. After incubation with 0.5% Triton X-100 and 2.5% BSA for 4 hours, the osteoblasts were stained with an antibody against tnni2 (Santa Cruz Biotechnology, USA) overnight at 4°C followed by incubation with a fluorescent antibody for 1 hour. DNA was stained using 1 µg/mL DAPI.

### BrdU incorporation assay

P12 and P13 heterozygous mutants and wild-type littermates were injected intraperitoneally with BrdU at 200 µg/g of body weight for 2 hours and were then sacrificed for BrdU staining (n = 4). BrdU labeled osteoblasts were assessed (BrdU positive osteoblast counts/area) using Image-Pro Plus 6.0 software.

### Vegf recovery experiment

A subcutaneous injection of recombinant mouse vegf165 (50 ng/g, PeproTech, USA) was performed on *Tnni2^+/K175del^* mice (n = 4) from day 4 to day 12. Heterozygous littermate controls were treated with NaCl (50 ng/g). Treated heterozygous mice and their heterozygous littermate controls were sacrificed at 13 days of age, and the radii and ulnae were collected for H&E staining. The trabecular bone density and number of osteoblasts was assessed using Image-Pro Plus 6.0 software. The trabecular bones/area was defined as the density of trabecular bones and the number of osteoblasts/density of trabecular bone was used to assess relative osteoblast number.

### Gene expression microarray analysis

Total RNA from the entire radii and ulnae of newborn *Tnni2^K175del/K175del^* mice (n = 3) and wild-type littermates (n = 3) was extracted using TRIZOL reagent. The mRNA was labeled, hybridized and scanned by CaptialBio (Beijing, China). Affymetrix Mouse Gene 1.0 ST Array was used to generate mRNA expression profile data. Differentially expressed genes associated with the *Tnni2^K175del^* mutation were analyzed using the SAM software 3.02. The GEO ID of the expression profile data is GSE32163. Gene enrichment analyses were performed on our Affymetrix Mouse Gene 1.0 ST microarray expression dataset of wild-type and mutant bones using GSEA 2.0 software [42]. An enrichment score and nominal *P* value were obtained for the genes that are be induced in human arterial endothelial cells following exposure to hypoxia condition [16].

### qPCR

Total RNA was extracted from the radii and ulnae of newborn *Tnni2^K175del/175K^* mice and wild-type littermates using TRIZOL reagent. Reverse transcription was performed using the M-MLV reverse system (Promega, USA). mRNA expression of *Hif*-associated signaling molecules, *Ihh*, *Pthrp*, *Col10a1*, *Comp*, *Ocn*, *SPP1* and *Ibsp* were quantified using qPCR. Data were analyzed using the 2(-Delta Delta C(T)) method.

### Chromatin immunoprecipitation (ChIP)-PCR

Primary osteoblasts from newborn *Tnni2^K175del/K175del^* mice (n = 3) and wild-type littermates (n = 3) were cultured as described above for ChIP-PCR. ChIP was performed as previously described [43]. Approximately 1×10^7^ osteoblasts from homozygous mice and wild-type littermates were prepared. Osteoblasts were cross-linked in 1% formaldehyde solution at 37°C for 10 min followed by 1/20 volume of 2.5 M glycine to stop the crosslinking. The cells were rinsed twice with ice-cold PBS, transferred to 15 mL conical centrifuge tubes, and spun for 4 min at 3000 rpm. Cell pellets were re-suspended in 10 mL of LB1 (50 mM Hepes–KOH, pH 7.5; 140 mM NaCl; 1 mM EDTA; 10% glycerol; 0.5% NP-40; 0.25% Triton X-100). The samples were rocked at 4°C for 10 min followed by a spin at 2000 rpm at 4°C for 4 min. The pellets were re-suspended in 10 mL of LB2 (10 mM Tris–HCl, pH 8.0; 200 mM NaCl; 1 mM EDTA; 0.5 mM EGTA). The samples were gently rocked at 4°C for 5 min. The nuclei were pelleted and collected by spinning at 2000 rpm at 4°C for 5 min. The pellets were resuspended in 3 mL LB3 (10 mM Tris–HCl, pH 8; 100 mM NaCl; 1 mM EDTA; 0.5 mM EGTA; 0.1% Na–deoxycholate; 0.5% N-lauroylsarcosine). The samples were sonicated using the following settings: 30 cycles of 15 s ON and 59 s OFF, power-output 31 watts. 10% Triton X-100 was added to sonicated lysate, followed by a 10 min spin at 12,000 rpm at 4°C. The supernatants were combined and eluted with 3-fold volume LB3. A subsample of 100 µL of cell lysate from sonication was saved as an input control and stored at −20°C. A total of 100 µL magnetic beads (Invitrogen, California, USA) was added to 30 µg of TNNI2 goat polyclonal IgG antibody (Santa Cruz,Biotechnology, USA) and 1 mg of goat serum, and incubated overnight at 4°C. The beads were washed twice in 1 mL block solution (0.5% BSA (w/v) in PBS). The beads were then collected using a magnetic stand and re-suspended in 100 µL block solution. A total of 100 µL of antibody/magnetic bead mix was added into cell lysates and gently mixed overnight at 4°C. The beads were collected with a magnetic stand and washed with 1 mL RIPA buffer (50 mM Hepes-KOH, pH 7.5; 500 mM LiCl; 1 mM EDTA; 1% NP-40; 0.7% Na-deoxycholate) four times, followed by one wash with 1 mL TBS (20 mM Tris-HCl, pH 7.6; 150 mM NaCl). The beads were then spun at 1500 rpm for 3 min at 4°C to remove any residual TBS buffer using the magnetic stand. A total of 200 µL of elution buffer (50 mM Tris-HCl, pH 8; 10 mM EDTA; 1% SDS) was added into each IP beads and 50 µL input control to reverse crosslink at 65°C for 16 hours. A total of 200 µL TE was added with 8 µL of 1 mg/mL RNaseA to each IP bead and input control DNA at 37°C for 30 min. A total of 4 µL 20 mg/mL proteinase K was added and incubated at 55°C for 2 hours. DNA was extracted with 400 µL phenol-chloroform isoamyl alcohol and precipitated DNA with cold ethanol. PCR was performed to test potent tnni2 binding sequences. PCR reactions contained 0.15 ng IP DNA and input control DNA. The primers for PCR reactions were shown in Table S3. The PCR products were tested using electrophoresis and Sanger sequencing.

### Establishing 293T stable cell lines that constitutively express wild-type tnni2-GFP and mutated tnni2-GFP

cDNA fragments of wild-type and *Tnni2^K175del^* mutation were subcloned into an EGFP-N1 vector to produce wild-type tnni2-GFP and mutated tnni2-GFP proteins, respectively. 293T cells were transfected with 20 µg mutated vector and the same amount of *Tnni2* wild-type vector. 1 mg/mL of G418 was used to screen anti-G418 cells for 2 weeks. GFP-positive cells were sorted by Flow Cytometry.

### Luciferase reporter assays

Wild-type and mutated *Tnni2* cDNA fragments were subcloned into pcDNA 3.1 vector. The truncated fragment (−1673 to 0 *Hif3a* promoter), the whole length fragment (−2200 to 0 *Hif3a* promoter) and the Part 1 fragment (−2200 to −1674 *Hif3a* promoter) were obtained by PCR amplification from mouse genomic DNA and subcloned into pGL3 luciferase reporter vector. 293T cells were respectively transfected with 100 ng mutated tnni2^K175del^ vector, wild-type tnni2 vector, hif3a (−1673 to 0) pGL3 vector, hif3a (−2200 to 0) pGL3 vector, hif3a (−2200 to −1674) pGL3 vector and pEGFP vector (as negative control), combined with 5 ng pRL-TK reporter vector and incubated under 1% or 20% O_2_ conditions for 30 hours. Dual reporter experiments were performed using the Dual-Glo luciferase assay system (Promega, Wisconsin, USA). The luminescence signals were detected using a GloMax-96 Microplate Luminometer (Promega, Wisconsin, USA). Firefly Luciferase was measured 30 hours later and normalized by Renilla Luciferase (pRP-TK) for transfection efficiency.

### Immunoprecipitation (IP) and mass spectrometry

Total cytoplasm extracts (CE) and nuclear extracts (NE) from 293T tnni2 wild-type-GFP stable cells were prepared according to the following method: Buffer A (10 mM HEPES, pH 7.9, 1 M HEPES, 10 mM KCL, 2 M KCL, 0.1 mM EDTA, 0.5 M EDTA, 0.1 mM EGTA) was added with 1 mM PMSF and 1 mM DTT into the cell pellet and then suspension was vortexed at 4°C for 10 min. After a spin at 3000 rpm for 5 min at 4°C, supernatant (CE) was collected and 5-fold volume buffer C (10 mM HEPES pH 7.9, 500 mM NaCl, 0.1 mM EDTA, 0.1 mM EGTA, 0.1% NP40, 10% glycerol) was added with 1 mM PMSF and 1 mM DTT to isolate the nuclear extract. The lysate was rotated for 20 min at 4°C and centrifuged at 12,000 rpm for 20 min at 4°C. The supernatant (NE) was transferred to a new tube. 20 mg of CE or NE was incubated with 200 µL agarose beads (Invitrogen, California, USA) that were coupled with an antibody against GFP protein at 4°C for 4 hours, followed by a spin at 1000 rpm for 5 min at 4°C. The supernatant was removed, and the agarose beads were resuspended with 1 mL Buffer C three times. The agarose beads were boiled in 1× protein loading buffer at 95°C for 30 min to separate the protein from the beads. After centrifugation at 12,000 rpm for 20 min at room temperature, the supernatant was collected. The IP products were used for mass spectrometry analyses.

### Electrophoretic mobility shift assay (EMSA)

Nuclear extracts of wild-type tnni2-GFP 293T stable cell lines and tnni2^K175del^-GFP stable cell lines were prepared as described above. The protein-DNA binding reactions were performed using the Thermo Scientific LightShift Chemiluminescent EMSA Kit. 20 mg of nuclear extract and 10 fmol of biotin-labeled probe were used for each binding reaction. For the competition assay, 5 pmol of cold probe was incubated with the samples at room temperature for 10 min prior to the addition of biotin-labeled probes. For the super-shift assay, 2 µg anti-GFP monoclonal antibody (Sigma) was used for each reaction. Incubated samples were separated by 6% non-denaturing PAGE at 100 V on ice for 80 min and transferred to a nitrocellulose filter membrane at 350 mA on ice for 40 min. The sequences of the 5′ biotin-labeled probe used for EMSA and Super-EMSA was: forward, 5′-ccctgtctagaatcccccagtgaggggctgggggcgtggctcagtggtagagc-3′, reverse, 5′-gctctaccactgagccacgcccccagcccctcactgggggattctagacaggg- 3′.

### DNA pull-down assay

10 mg of the 5′ biotin-labeled probe (the same as the probe used in EMSA assay) and 100 µL avidin magnetic beads (Invitrogen, Dynabeads) were added into a 1.5 mL centrifuge tube with 0.5 mL buffer C (refer to IP assays above) and incubated at room temperature for 1 hour. The beads were collected using a magnetic stand. 20 mg of NE from wild-type tnni2-GFP and tnni2^K175del^-GFP 293T stable lines were added into the centrifuge tubes with a magnetic bead-probe and they were incubated at room temperature for 1 hour. The supernatant was removed, and the magnetic beads were collected with a magnetic bead stand. The beads were carefully washed with buffer C three times. The magnetic beads were boiled using 80 µL 1×protein loading buffer at 95°C for 30 min to separate the protein from the beads. Following centrifugation at 12,000 rpm for 20 min at room temperature, the supernatant was collected. 40 mL of IP products from wild-type and mutated NE were examined by western blotting using an anti-GFP monoclonal antibody (Sigma). The products isolated from beads that were incubated only with wild-type and mutant NE were used as negative controls. The NE from wild-type TNNI2-GFP were used as Input. The experiment was performed using four independent biological repeats.

### ELISA

Protein concentrations of soluble vegf were determined using the Valukine ELISA kit for mouse VEGF (R&D Systems). Cell culture supernatants (*Tnni2^K175del/K175del^* and wild type primary osteoblast cells) from triplicates of three different experiments were harvested after 18 hours, 24 hours and 36 hours respectively, centrifuged with 12,000 rpm for 5 min and stored at −20°C. Each corresponding well was subsequently trypsinized and the numbers of live cells were counted to permit the appropriate correction of secreted vegf. ELISA assays were performed according to the manufacturer's instructions.

### Cell proliferation assays

Cell proliferation assays were performed with a CellTiter 96 AQueous Proliferation Assay kit (Promega, Wisconsin, USA) following the manufacturer's instructions, and the plates were measured with a Molecular Devices plate reader.

### Western blotting

Proteins were separated by SDS-PAGE and transferred to a PVDF membrane (Millipore, Billerica, USA). The membrane was blocked with 5% non-fat milk and incubated with rabbit anti-HIF3A (1/500, Abcam Biotech, Cambridge, UK), rabbit anti-HIF1A (1/500, Abcam Biotech, Cambridge, UK), rabbit anti-VEGF (1/1000, Abcam Biotech, Cambridge, UK), rabbit anti-TNNI2 (1/500, Merck Chemicals, Nottingham, UK), Rabbit ant-histone H3 (1/800, Cell Signaling Technology, Danvers, USA) or mouse anti-β-actin (1/10000, Sigma, St Louis, USA) antibodies.

### Statistical analysis

Data were processed using SPSS 17.0 and denoted as the mean±s.d. A comparison between the quantitative data of the two groups was performed using Student's *t*-test (two-tailed distribution). Statistical significance was indicated by *P*<0.05.

## Supporting Information

Figure S1 Generation and detection of *Tnni2^K175del^* knock-in mice. (A) Genotypes detected using PCR. (B) The del-aag(175K) was deleted from the genome. (C) The del-aag(175K) was detected at the mRNA level using Sanger sequencing. (D) Southern blot analysis showed that the genomic DNA samples were prepared from *Tnni2^K175del/K175del^*, *Tnni2^+/K175del^*, and wild-type mice, digested with *EcoR* V, and probed to produce 5.6 kb and 16 kb bands that corresponded to the mutant and wild-type genotypes, respectively. (E, F) Schematic diagram of the homologous recombination of *Tnni2^K175del^* targeting vector and the southern blot test.
(JPG)

Figure S2 (A–C) *Tnni2^+/K175del^* and *Tnni2^K175del/K175del^* mice exhibited contracture phenotypes in forelimbs (*arrow*). (B) *Tnni2^K175del/K175del^* mice showed remarkably smaller compared to wild-type littermate at 1-day-old age.
(JPG)

Figure S3 *Tnni2^K175del^* mutant mice showed no significant differences in body size and weight relative to their wild-type littermates before E 18.5. (A) Neither heterozygous nor homozygous embryos displayed differences in body size compared to wild-type controls. (B) The comparison of weights of embryos from E15.5 to E18.5.
(JPG)

Figure S4 Survival curve of *Tnni2^+/K175del^* and *Tnni2^K175del/K175del^* mutants and wild-type littermates (n = 467). All homozygous mutants died before weaning.
(JPG)

Figure S5 (A–G) In situ hybridization analyses with antisense *Tnni2* riboprobe on histological sections of wild-type brains, hearts, esophagus, lungs, stomachs, spleens, skeletal muscles, kidneys from E15.5 embryos. mRNA of *Tnni2* was showed as pink color. Scale bar, 100 µm (A–G).
(JPG)

Figure S6 *Tnni2* was expressed in chondrocytes and osteoblasts of mutant mice. (A–C) The expression of tnni2 in the growth plates of radii and ulnae from *Tnni2^K175del/K175del^* mice exhibited a spatial and temporal change. (A) In E15.5 mutant embryos, tnni2 was expressed in pre-hypertrophic and hypertrophic chondrocytes (*arrows*). (B) At E17.5 wild-type embryos, tnni2 was observed in proliferating chondrocytes (*arrow*). (C) At P6 homozygous mutant radii, expression of tnni2 was in pre-hypertrophic, hypertrophic, immature proliferating and rest chondrocytes (*arrows*), whereas no discernible tnni2 was observed in mature proliferative chondrocytes. (D) Expression of tnni2 was in articular chondrocytes of tibiae of P13 *Tnni2^K175del/K175del^* mice (*arrow*). (E) tnni2 was observed in osteoblasts of E18.5 homozygous mutant mice (*arrows*). (Scale bar, 100 µm (A–E). Embryonic day, E.
(JPG)

Figure S7 *Left panel:* Von Kossa staining of non-demineralized calvaria of heterozygous mutant and their wild-type mice at P2 (n = 3). *Right panel*: Quantification analyses of mineralization in heterozygous mutant and wild-type calvaria. Student's *t*-test, mean±s.d.
(JPG)

Figure S8 Quantification analyses of the first ossification center lengths of radii, ulnae, humeri and scapulae at E14.75 homozygous mutants and wild-type littermates (n = 2).
(JPG)

Figure S9 qPCR analyses assessed the mRNA expression of *Hif1a*, *Hif3a*, *Vegf^164/188^* and *Flt1* (normalized to *Gapdh*) in the calvariae from 1-day old *Tnni2^K175del/K175del^* mice and wild-type littermates. Student's *t*-test, mean±s.d.
(JPG)

Figure S10 Von Kossa assay stained mineralized cartilage matrix and trabecular bones at 1-day old growth plate of radii from *Tnni2^K175del/K175del^* mice and wild-type littermates. Mineralized cartilage matrix (black) was markedly decreased and the more number of hypertrophic cells was observed in homozygous mutants compared to wild-type littermates. Scale bar, 100 µm.
(JPG)

Figure S11 Alkaline phosphatase staining revealed no differences in differentiation of the primary osteoblasts between mutants and wild-type littermates *in vitro* cultures (n = 3).
(JPG)

Figure S12 Western blot analyses of tnni2 expression in nuclear protein extract from primary wild-type osteoblasts under hypoxic and normoxic conditions (n = 3).
(JPG)

Figure S13 Immunofluorescence staining of tnni2 (*arrows*) in the cytoplasm and nucleus of the primary osteoblasts from newborn *Tnni2^K175del/K175del^* mutant mice under both hypoxic (1% O_2_) and normoxic (20% O_2_) conditions. The mutant tnni2 was stained as green. Nuclei were stained with DAPI (blue). Scale bar, 100 µm (A and B).
(JPG)

Figure S14 tnni2 located at the nucleus of 293T cells and HepG2 cells. 293T cells (A) and HepG2 cells (B) marked by tnni2-GFP showed that tnni2 was observed in the nucleus under 1% and 20% oxygen conditions. Nuclei were stained with DAPI (blue). *Arrows* indicated tnni2-GFP. Scale bar, 100 µm (A and B).
(JPG)

Figure S15 Luciferase assays performed using 293T cell line under 1% or 20% O_2_ conditions. Firefly Luciferase was measured and normalized to Renilla Luciferase (pRP-TK). Ratio of the normalized Firefly Luciferase expression to that of control vector was used to represent relative Luciferase activity. Student's *t*-test. Data represents the mean±s.d. of eight experiments carried out in quadruplicate.
(JPG)

Figure S16 Western blot analyses of tnni2, hif3a, vegf and hif1a in primary chondrocytes from newborn heterozygous mutants and wild-type littermate mice under 1% O_2_ condition.
(TIF)

Figure S17 qPCR analyzed the relative mRNA expression of *Ocn*, *Spp1* and *Ibsp* (normalized to *Gapdh*) in primary wild-type and homozygous mutant osteoblasts (Home/WT) that were cultured under 10 mM β-glycerophosphate and 50 µg/mL ascorbic acid for 14 days.
(JPG)

Figure S18 Activation of osteoclasts had no difference between homozygous mutant mice and wild-type littermates. (A) Tartrate-resistant acid phosphatase (TRAP) staining showed activated osteoclasts at E16.5 homozygous mutant radii (n = 3) and wild-type controls (n = 3). Osteoclasts were stained as violet (*arrows*). (B) In situ hybridization analyses with antisense *Mmp9* riboprobe on histological sections of radii and ulnae from E17.5 homozygous mutant embryos (n = 2) and wild-type controls (n = 2). (C) qPCR analyses assessed the mRNA level of *Mmp9* and *Trap* (normalized to *Gapdh*) in the radii and ulnae from newborn *Tnni2^K175del/K175del^* mice and wild-type littermates. mean±s.d., Scale bar, 100 µm (A, B).
(JPG)

Figure S19 The expression level of *Runx2*. (A) Immunostaining analyses showed that the runx2 protein had no significant difference at hypertrophic cell zones in radii of E16.5 homozygous mutants and wild-type littermates (n = 3). (B) Microarray expression data of *Runx2* in the radii and ulnae of newborn *Tnni2^K175del/K175del^* mice (n = 3) and wild-type littermates (n = 3). Student's *t*-test, mean±s.d., Scale bar, 100 µm (A)
(JPG)

Table S1 The summary of significantly differential expression genes in the microarray dataset.
(PDF)

Table S2 Immunoprecipitation and mass spectrometry analyses revealed potential proteins interaction with tnni2 using the nuclear extracts from 293T cells ectopically expressing wild-type tnni2-GFP fusion protein. KPNB1, IPO5, IPO8, IPO9, TNPO1 and TNPO2 were highlighted in red. TNNI2 was marked in bold.
(PDF)

Table S3 Primers for ChIP-PCR.
(PDF)
