# Supplementary figures and images for: A Gain-of-Function Mutation in Tnni2 Impeded Bone Development through Increasing Hif3a Expression in DA2B Mice
Source: PLoS Genet. 2014 Oct 23;10(10):e1004589. doi: 10.1371/journal.pgen.1004589 (PMC4207604; doi:10.1371/journal.pgen.1004589)

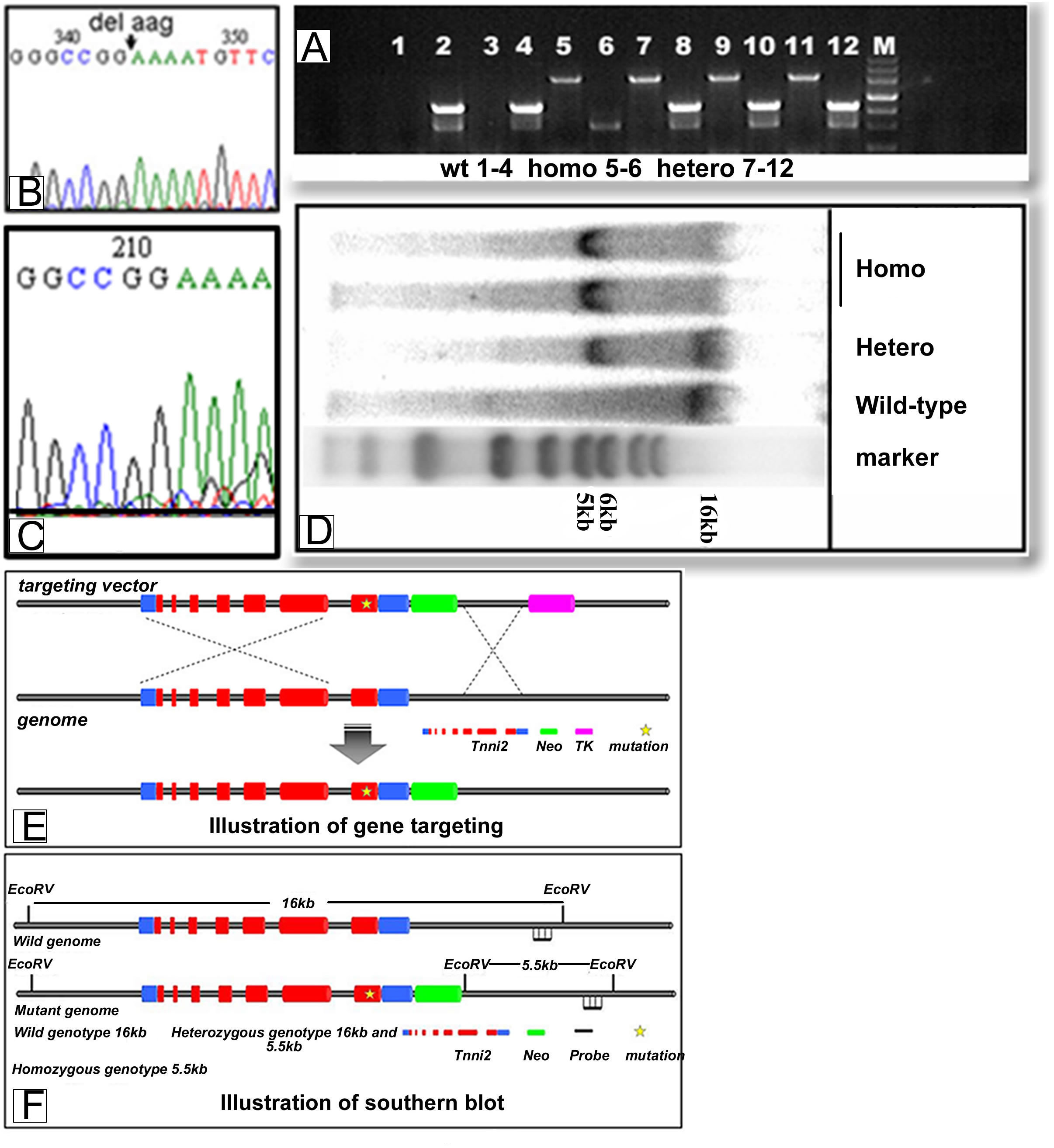

Supplement: Figure S1 — Generation and detection of Tnni2K175del knock-in mice. (A) Genotypes detected using PCR. (B) The del-aag(175K) was deleted from the genome. (C) The del-aag(175K) was detected at the mRNA level using Sanger sequencing. (D) Southern blot analysis showed that the genomic DNA samples were prepared from Tnni2K175del/K175del, Tnni2+/K175del, and wild-type mice, digested with EcoR V, and probed to produce 5.6 kb and 16 kb bands that corresponded to the mutant and wild-type genotypes, respectively. (E, F) Schematic diagram of the homologous recombination of Tnni2K175del targeting vector and the southern blot test. (JPG) [file pgen.1004589.s001.jpg]

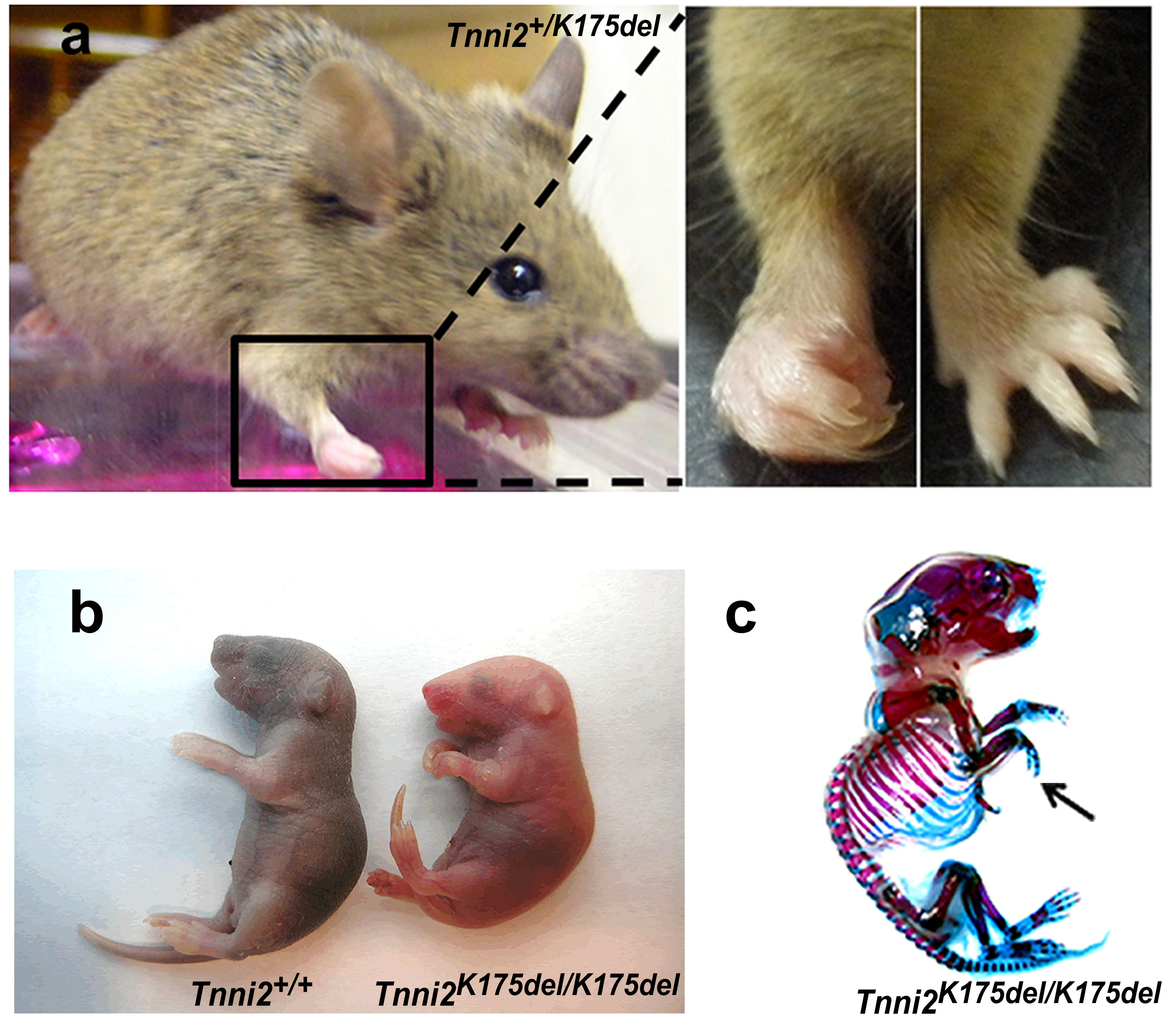

Supplement: Figure S2 — (A–C) Tnni2+/K175del and Tnni2K175del/K175del mice exhibited contracture phenotypes in forelimbs (arrow). (B) Tnni2K175del/K175del mice showed remarkably smaller compared to wild-type littermate at 1-day-old age. (JPG) [file pgen.1004589.s002.jpg]

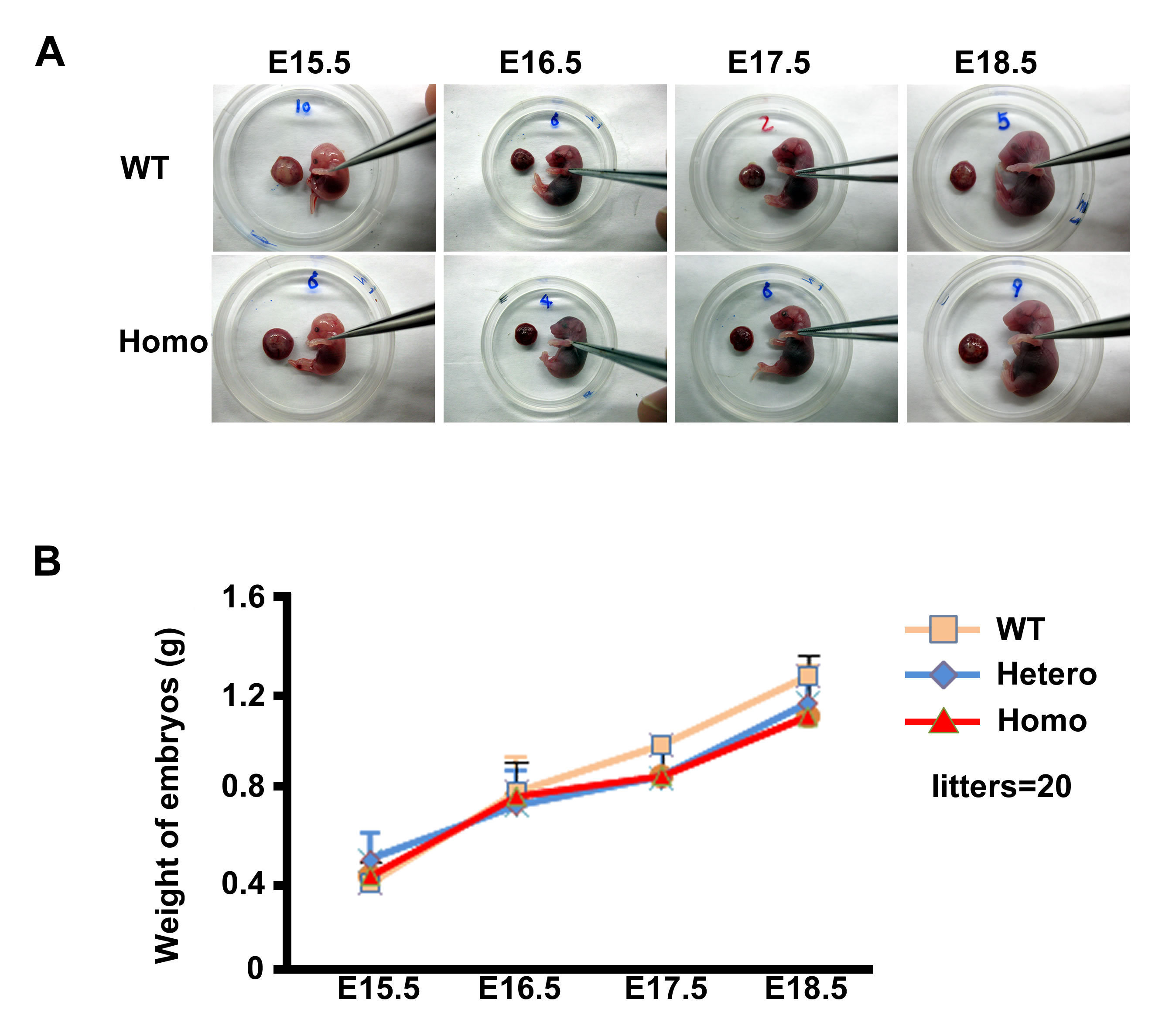

Supplement: Figure S3 — Tnni2K175del mutant mice showed no significant differences in body size and weight relative to their wild-type littermates before E 18.5. (A) Neither heterozygous nor homozygous embryos displayed differences in body size compared to wild-type controls. (B) The comparison of weights of embryos from E15.5 to E18.5. (JPG) [file pgen.1004589.s003.jpg]

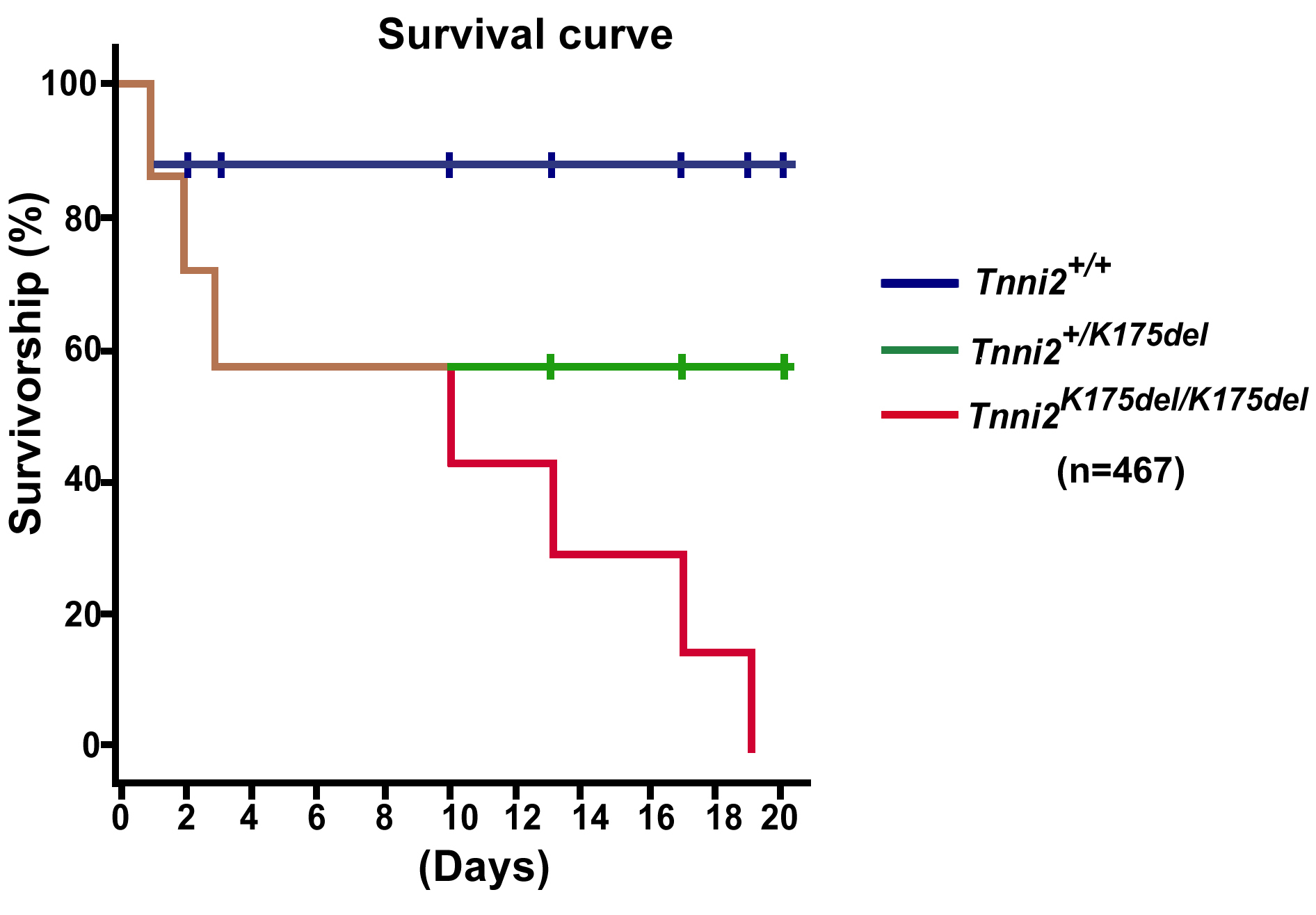

Supplement: Figure S4 — Survival curve of Tnni2+/K175del and Tnni2K175del/K175del mutants and wild-type littermates (n = 467). All homozygous mutants died before weaning. (JPG) [file pgen.1004589.s004.jpg]

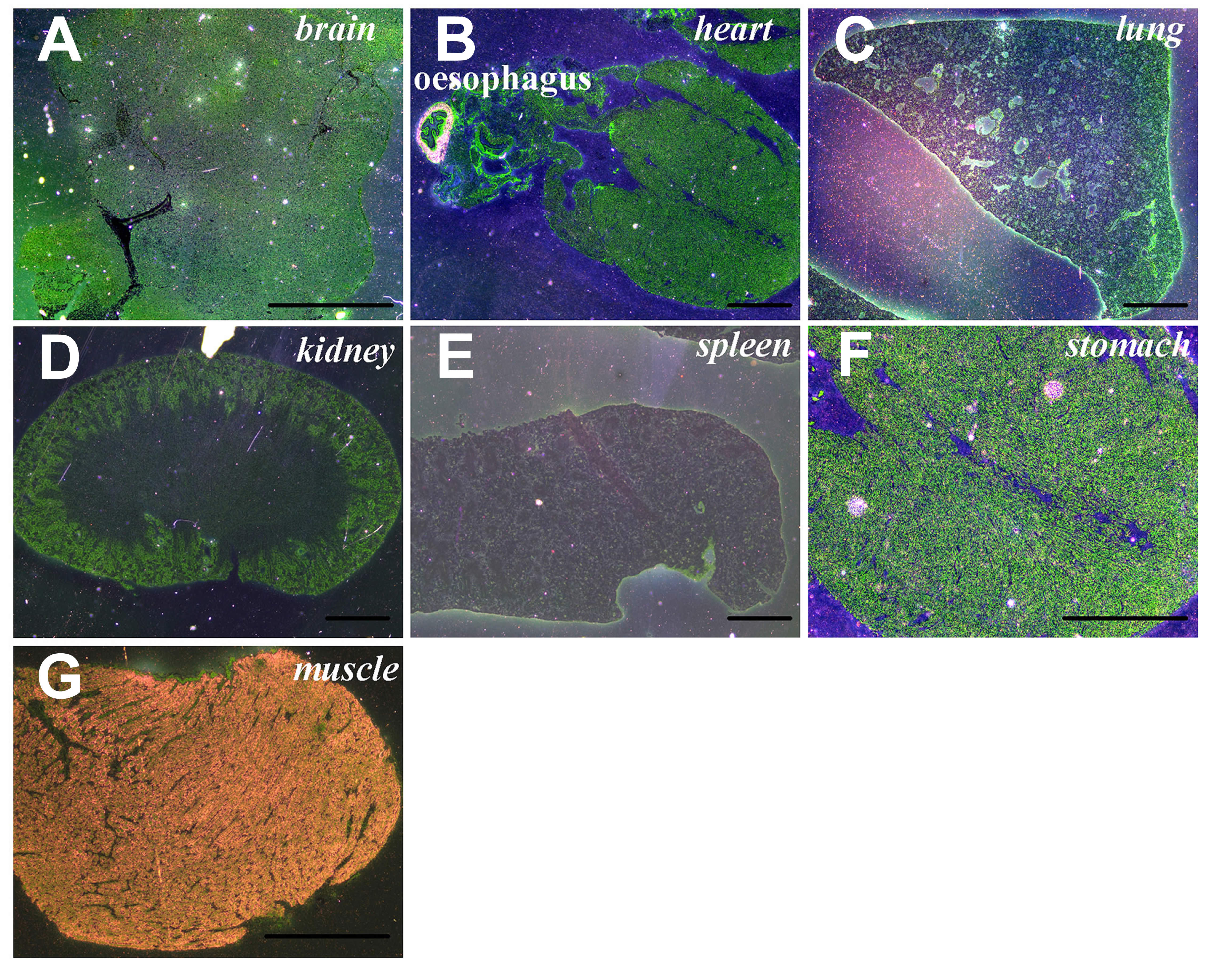

Supplement: Figure S5 — (A–G) In situ hybridization analyses with antisense Tnni2 riboprobe on histological sections of wild-type brains, hearts, esophagus, lungs, stomachs, spleens, skeletal muscles, kidneys from E15.5 embryos. mRNA of Tnni2 was showed as pink color. Scale bar, 100 µm (A–G). (JPG) [file pgen.1004589.s005.jpg]

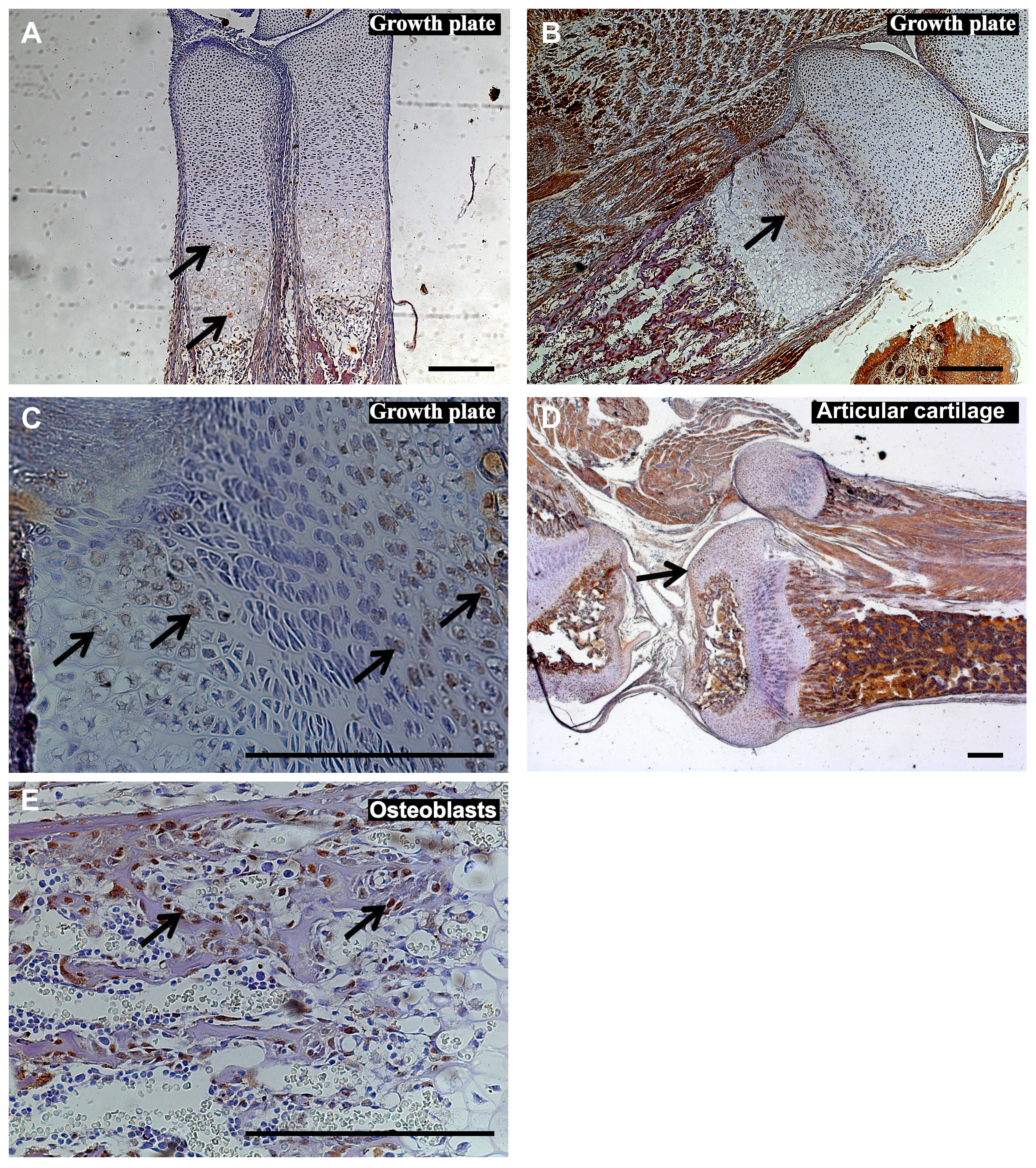

Supplement: Figure S6 — Tnni2 was expressed in chondrocytes and osteoblasts of mutant mice. (A–C) The expression of tnni2 in the growth plates of radii and ulnae from Tnni2K175del/K175del mice exhibited a spatial and temporal change. (A) In E15.5 mutant embryos, tnni2 was expressed in pre-hypertrophic and hypertrophic chondrocytes (arrows). (B) At E17.5 wild-type embryos, tnni2 was observed in proliferating chondrocytes (arrow). (C) At P6 homozygous mutant radii, expression of tnni2 was in pre-hypertrophic, hypertrophic, immature proliferating and rest chondrocytes (arrows), whereas no discernible tnni2 was observed in mature proliferative chondrocytes. (D) Expression of tnni2 was in articular chondrocytes of tibiae of P13 Tnni2K175del/K175del mice (arrow). (E) tnni2 was observed in osteoblasts of E18.5 homozygous mutant mice (arrows). (Scale bar, 100 µm (A–E). Embryonic day, E. (JPG) [file pgen.1004589.s006.jpg]

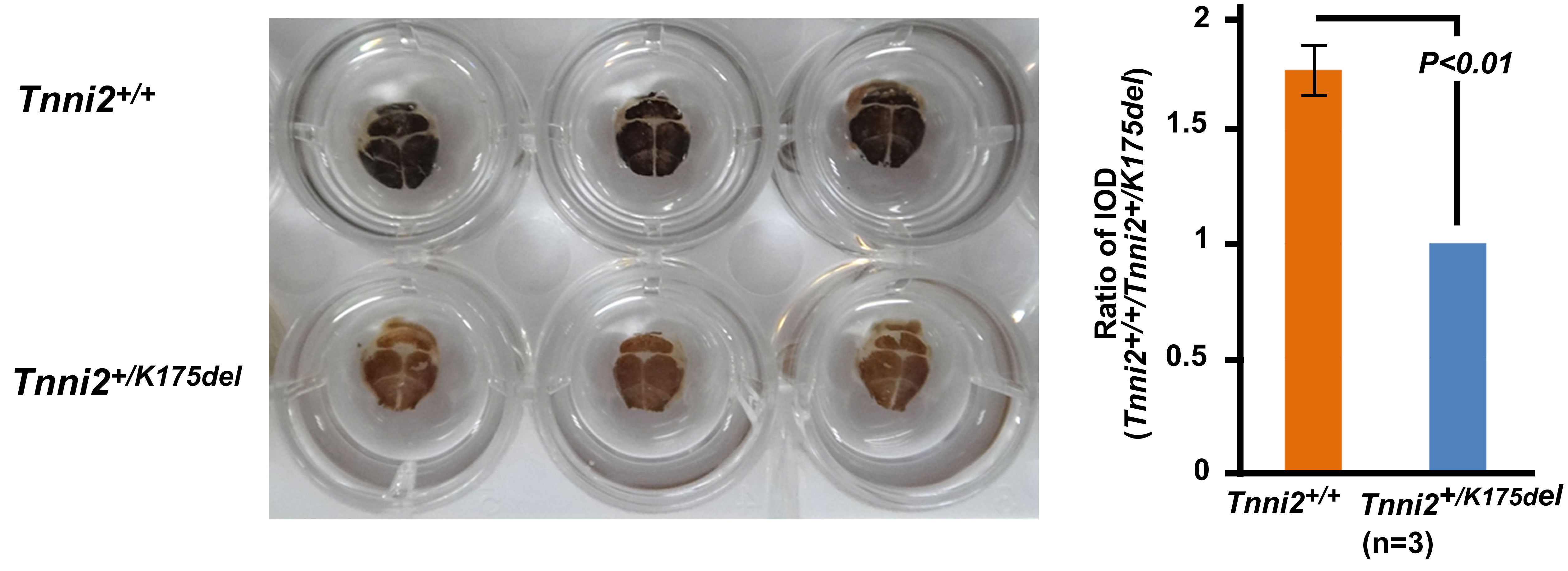

Supplement: Figure S7 — Left panel: Von Kossa staining of non-demineralized calvaria of heterozygous mutant and their wild-type mice at P2 (n = 3). Right panel: Quantification analyses of mineralization in heterozygous mutant and wild-type calvaria. Student's t-test, mean±s.d. (JPG) [file pgen.1004589.s007.jpg]

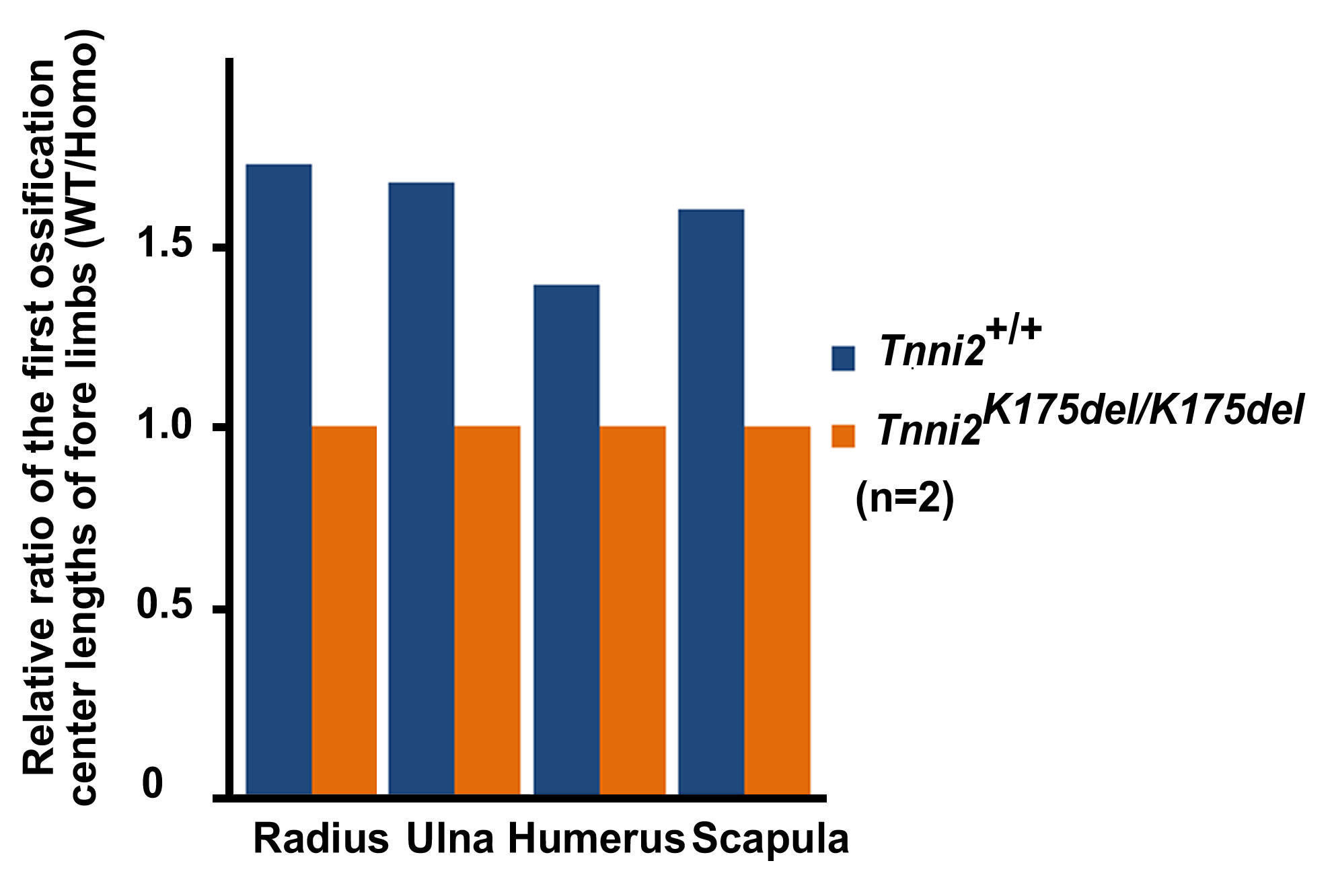

Supplement: Figure S8 — Quantification analyses of the first ossification center lengths of radii, ulnae, humeri and scapulae at E14.75 homozygous mutants and wild-type littermates (n = 2). (JPG) [file pgen.1004589.s008.jpg]

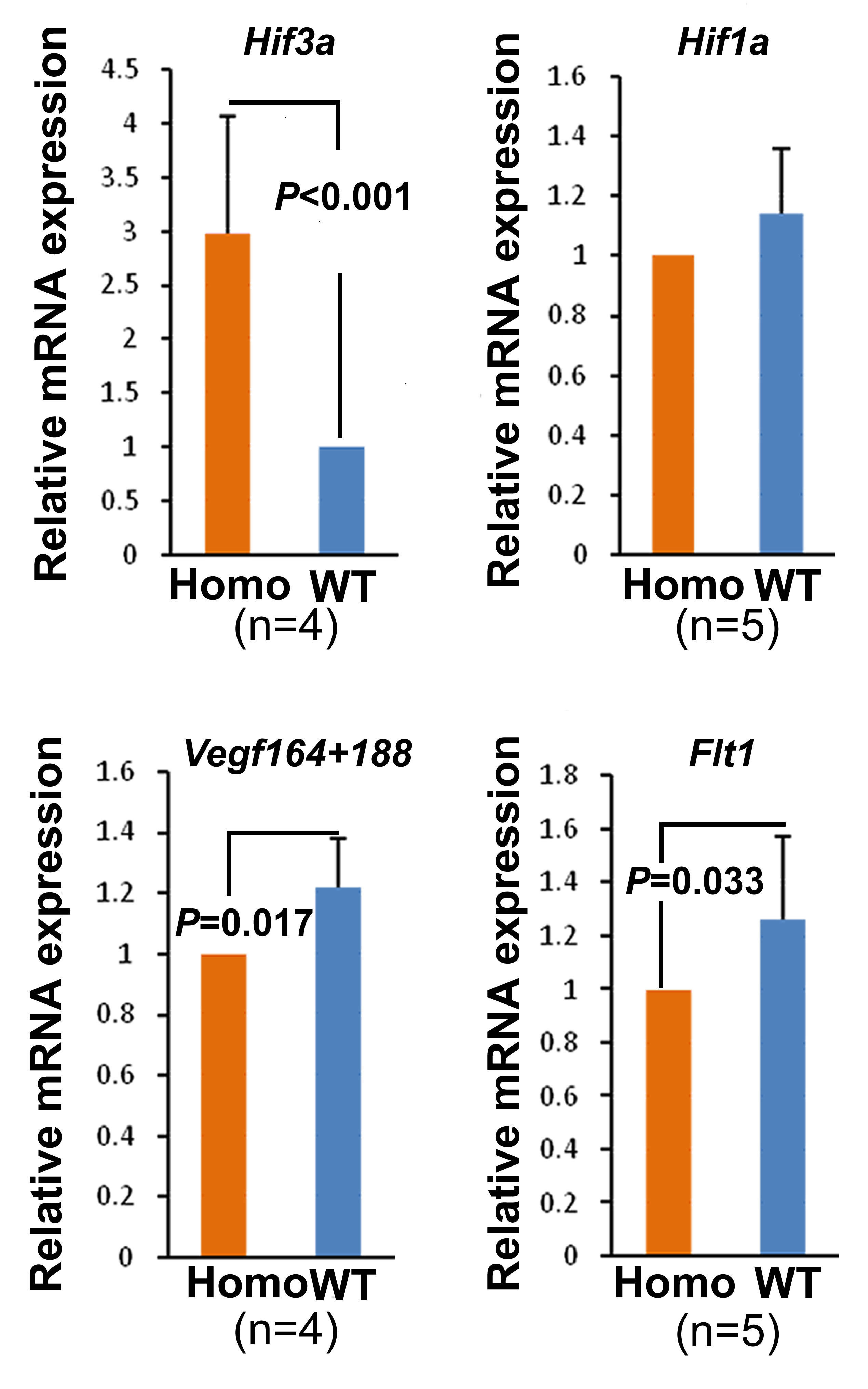

Supplement: Figure S9 — qPCR analyses assessed the mRNA expression of Hif1a, Hif3a, Vegf164/188 and Flt1 (normalized to Gapdh) in the calvariae from 1-day old Tnni2K175del/K175del mice and wild-type littermates. Student's t-test, mean±s.d. (JPG) [file pgen.1004589.s009.jpg]

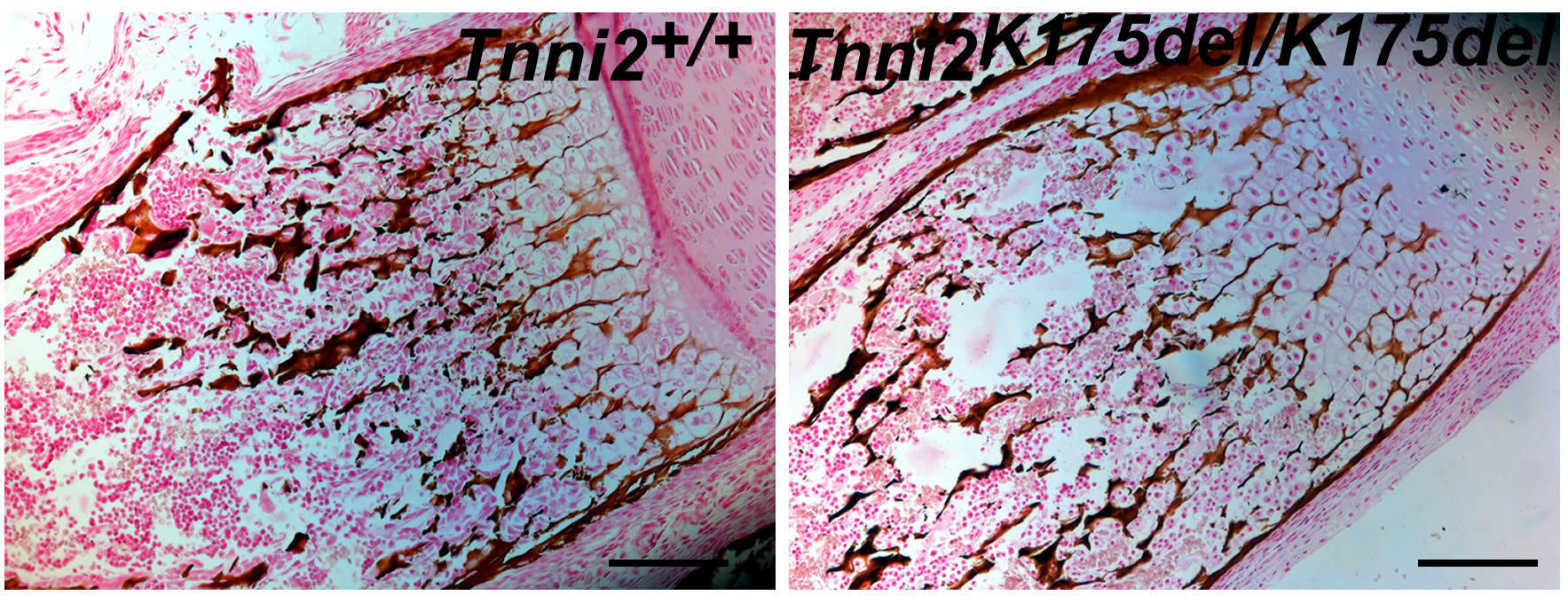

Supplement: Figure S10 — Von Kossa assay stained mineralized cartilage matrix and trabecular bones at 1-day old growth plate of radii from Tnni2K175del/K175del mice and wild-type littermates. Mineralized cartilage matrix (black) was markedly decreased and the more number of hypertrophic cells was observed in homozygous mutants compared to wild-type littermates. Scale bar, 100 µm. (JPG) [file pgen.1004589.s010.jpg]

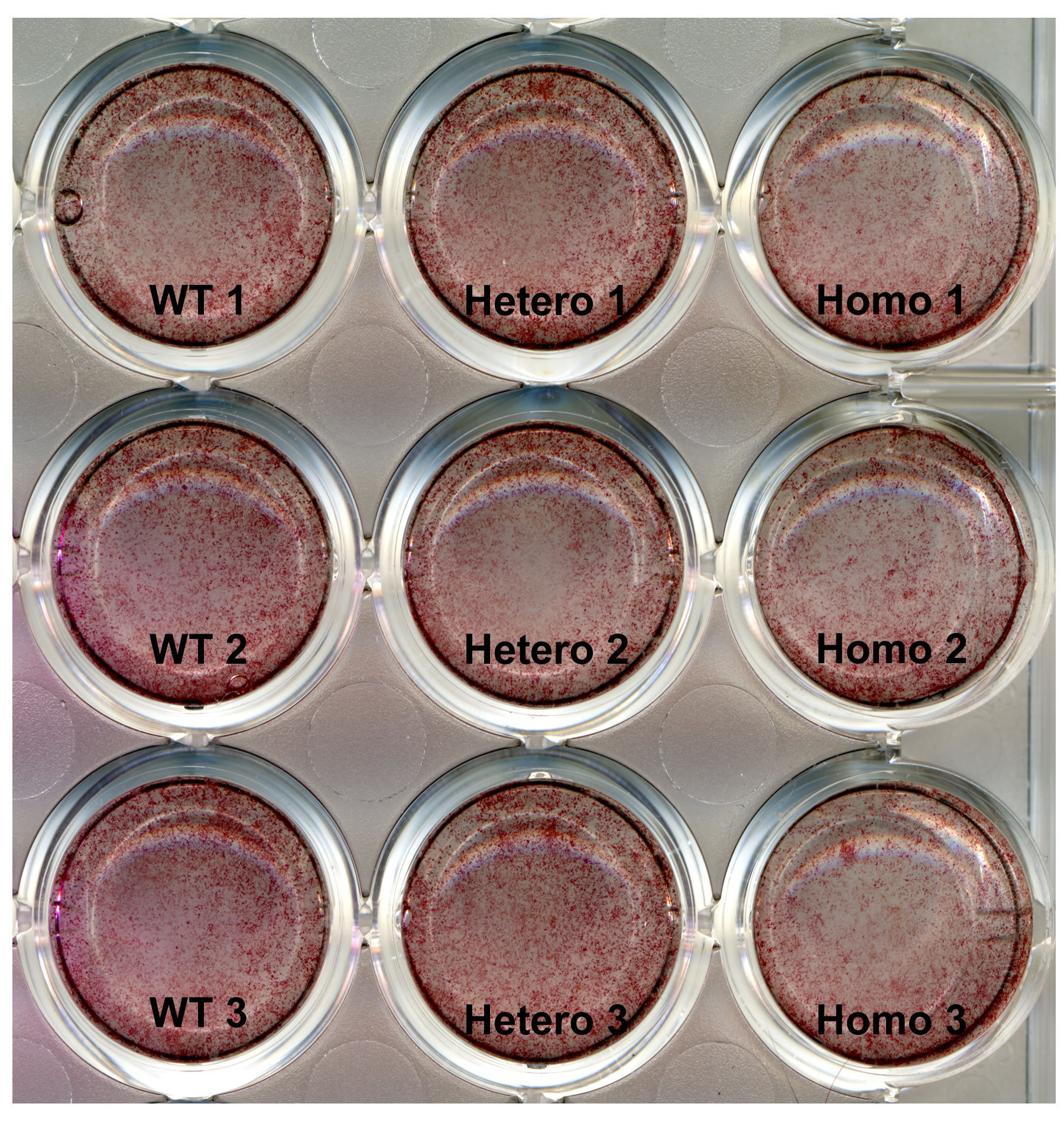

Supplement: Figure S11 — Alkaline phosphatase staining revealed no differences in differentiation of the primary osteoblasts between mutants and wild-type littermates in vitro cultures (n = 3). (JPG) [file pgen.1004589.s011.jpg]

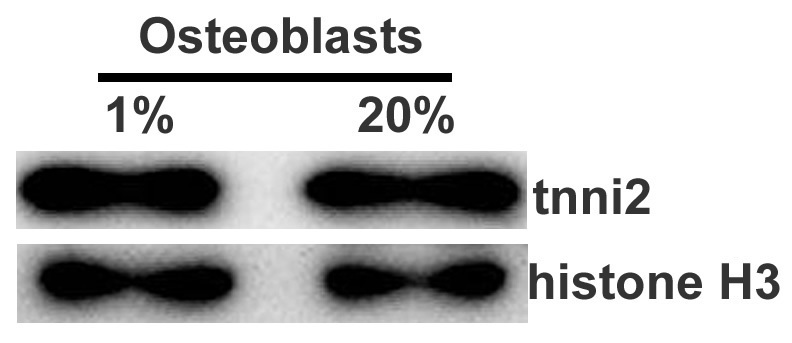

Supplement: Figure S12 — Western blot analyses of tnni2 expression in nuclear protein extract from primary wild-type osteoblasts under hypoxic and normoxic conditions (n = 3). (JPG) [file pgen.1004589.s012.jpg]

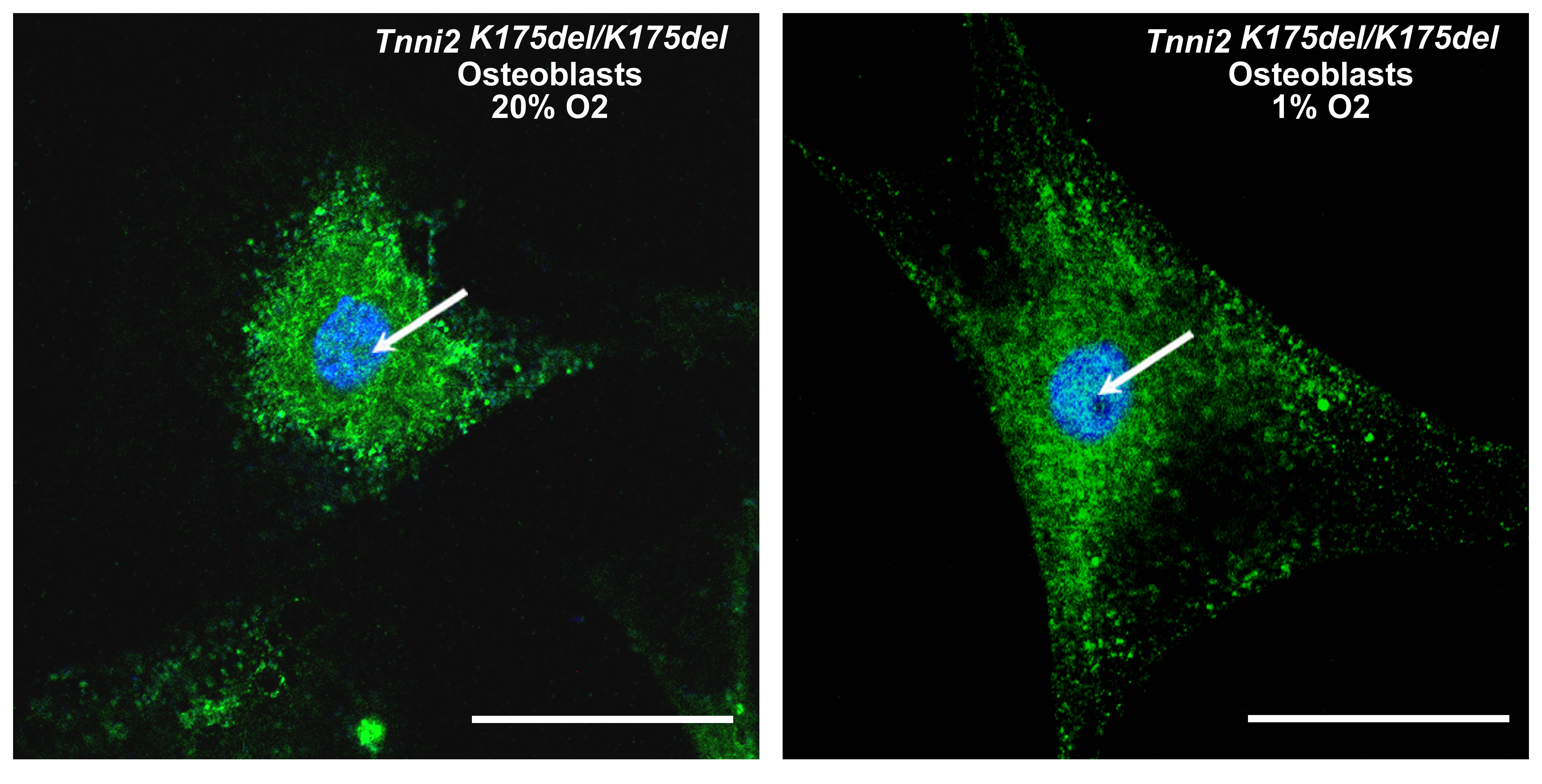

Supplement: Figure S13 — Immunofluorescence staining of tnni2 (arrows) in the cytoplasm and nucleus of the primary osteoblasts from newborn Tnni2K175del/K175del mutant mice under both hypoxic (1% O2) and normoxic (20% O2) conditions. The mutant tnni2 was stained as green. Nuclei were stained with DAPI (blue). Scale bar, 100 µm (A and B). (JPG) [file pgen.1004589.s013.jpg]

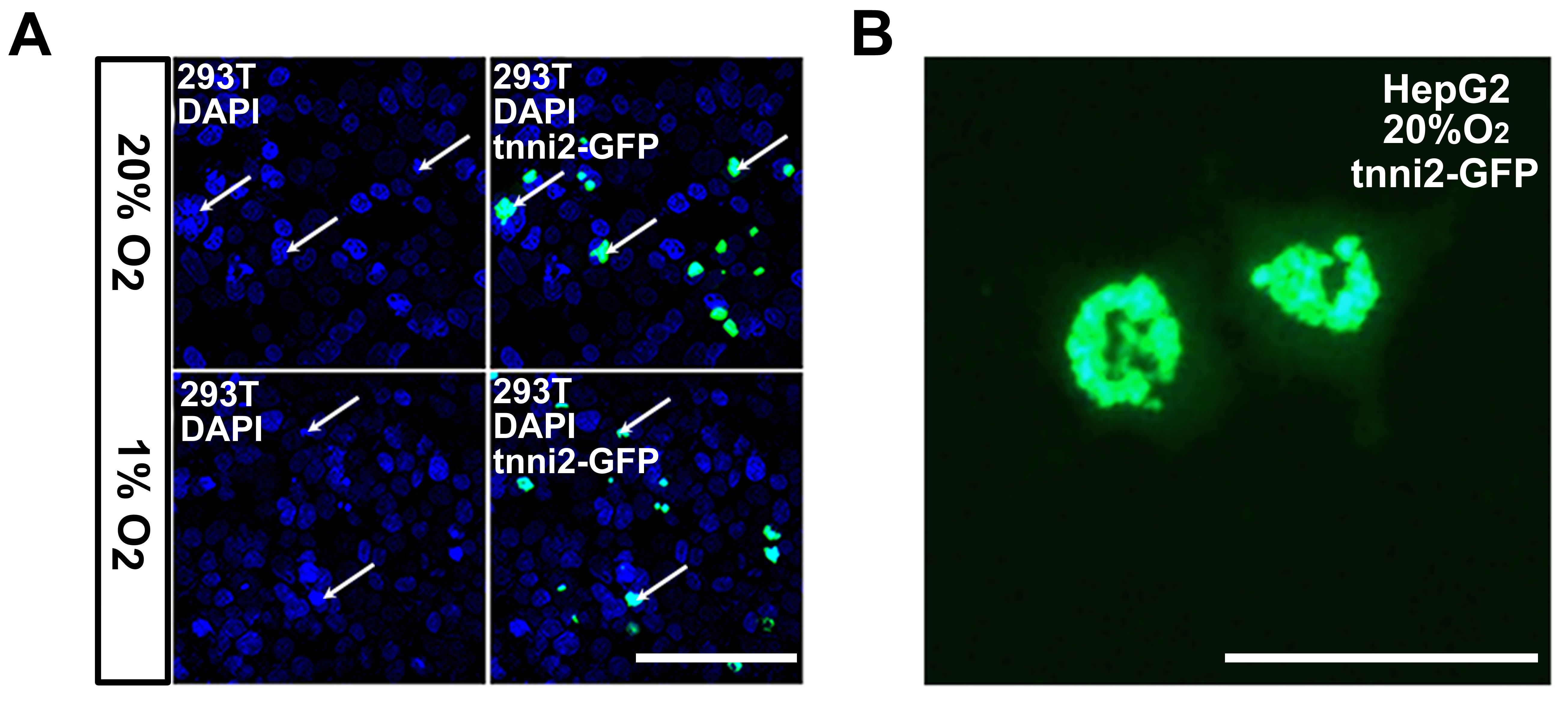

Supplement: Figure S14 — tnni2 located at the nucleus of 293T cells and HepG2 cells. 293T cells (A) and HepG2 cells (B) marked by tnni2-GFP showed that tnni2 was observed in the nucleus under 1% and 20% oxygen conditions. Nuclei were stained with DAPI (blue). Arrows indicated tnni2-GFP. Scale bar, 100 µm (A and B). (JPG) [file pgen.1004589.s014.jpg]

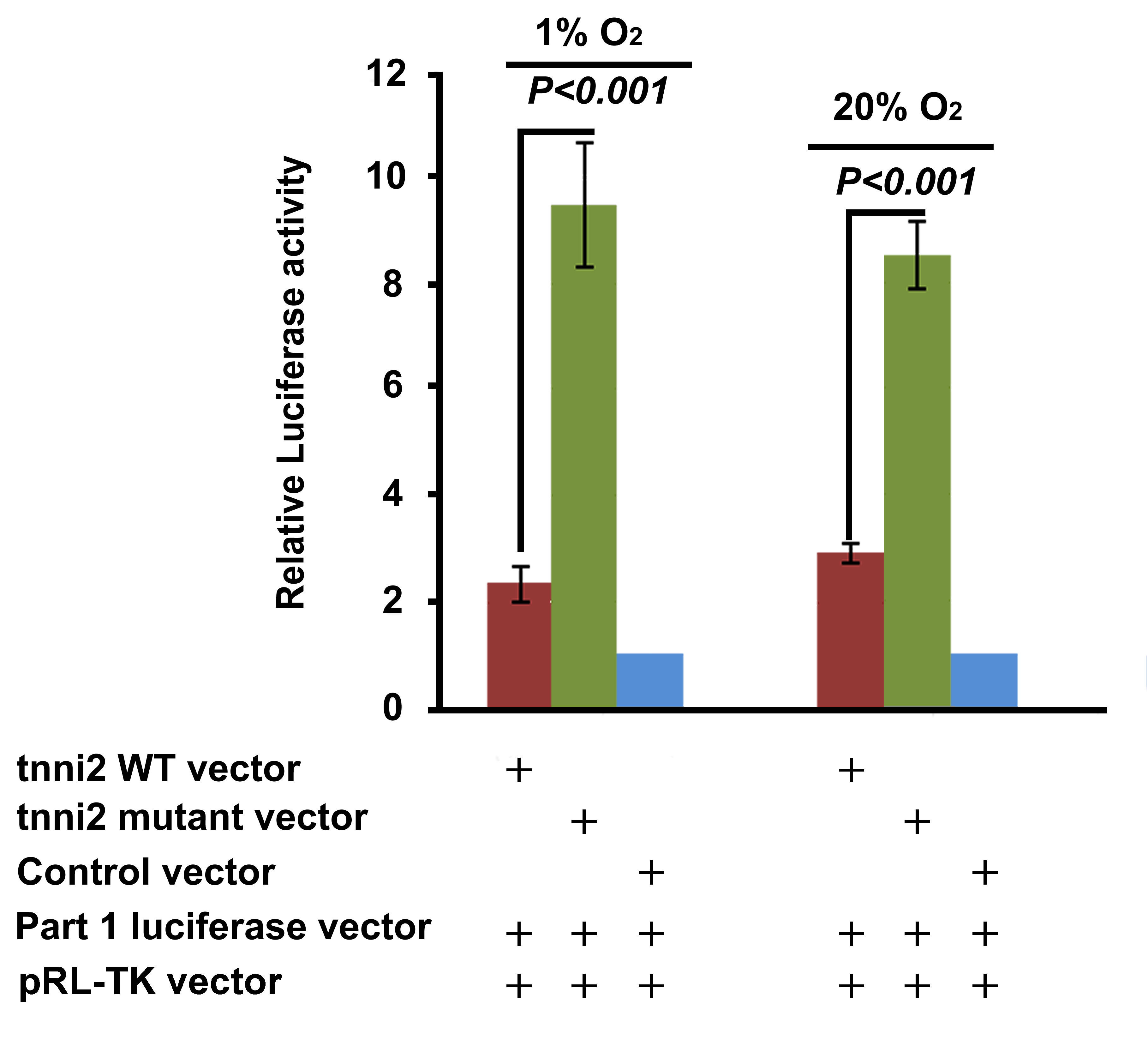

Supplement: Figure S15 — Luciferase assays performed using 293T cell line under 1% or 20% O2 conditions. Firefly Luciferase was measured and normalized to Renilla Luciferase (pRP-TK). Ratio of the normalized Firefly Luciferase expression to that of control vector was used to represent relative Luciferase activity. Student's t-test. Data represents the mean±s.d. of eight experiments carried out in quadruplicate. (JPG) [file pgen.1004589.s015.jpg]

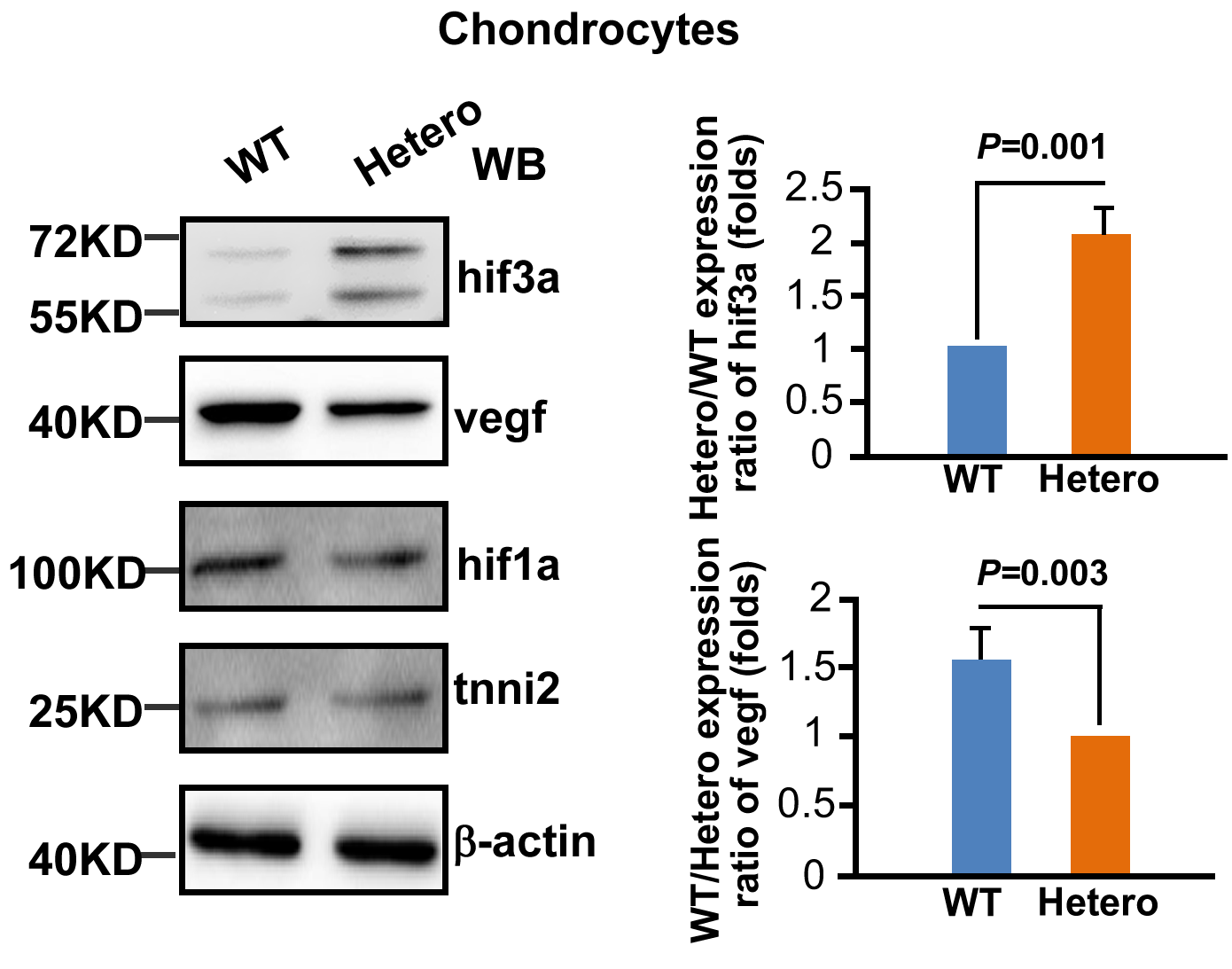

Supplement: Figure S16 — Western blot analyses of tnni2, hif3a, vegf and hif1a in primary chondrocytes from newborn heterozygous mutants and wild-type littermate mice under 1% O2 condition. (TIF) [file pgen.1004589.s016.tif]

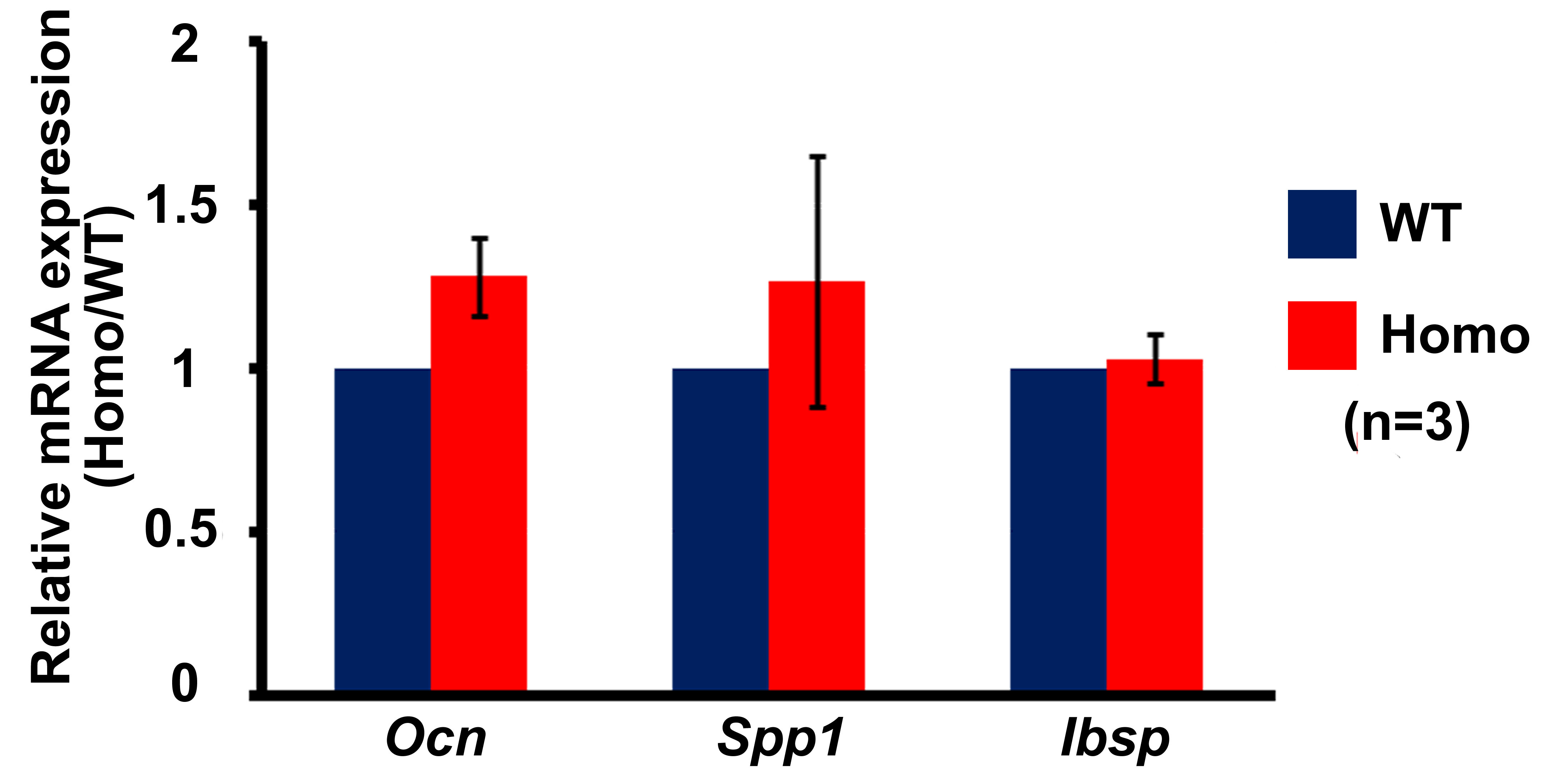

Supplement: Figure S17 — qPCR analyzed the relative mRNA expression of Ocn, Spp1 and Ibsp (normalized to Gapdh) in primary wild-type and homozygous mutant osteoblasts (Home/WT) that were cultured under 10 mM β-glycerophosphate and 50 µg/mL ascorbic acid for 14 days. (JPG) [file pgen.1004589.s017.jpg]

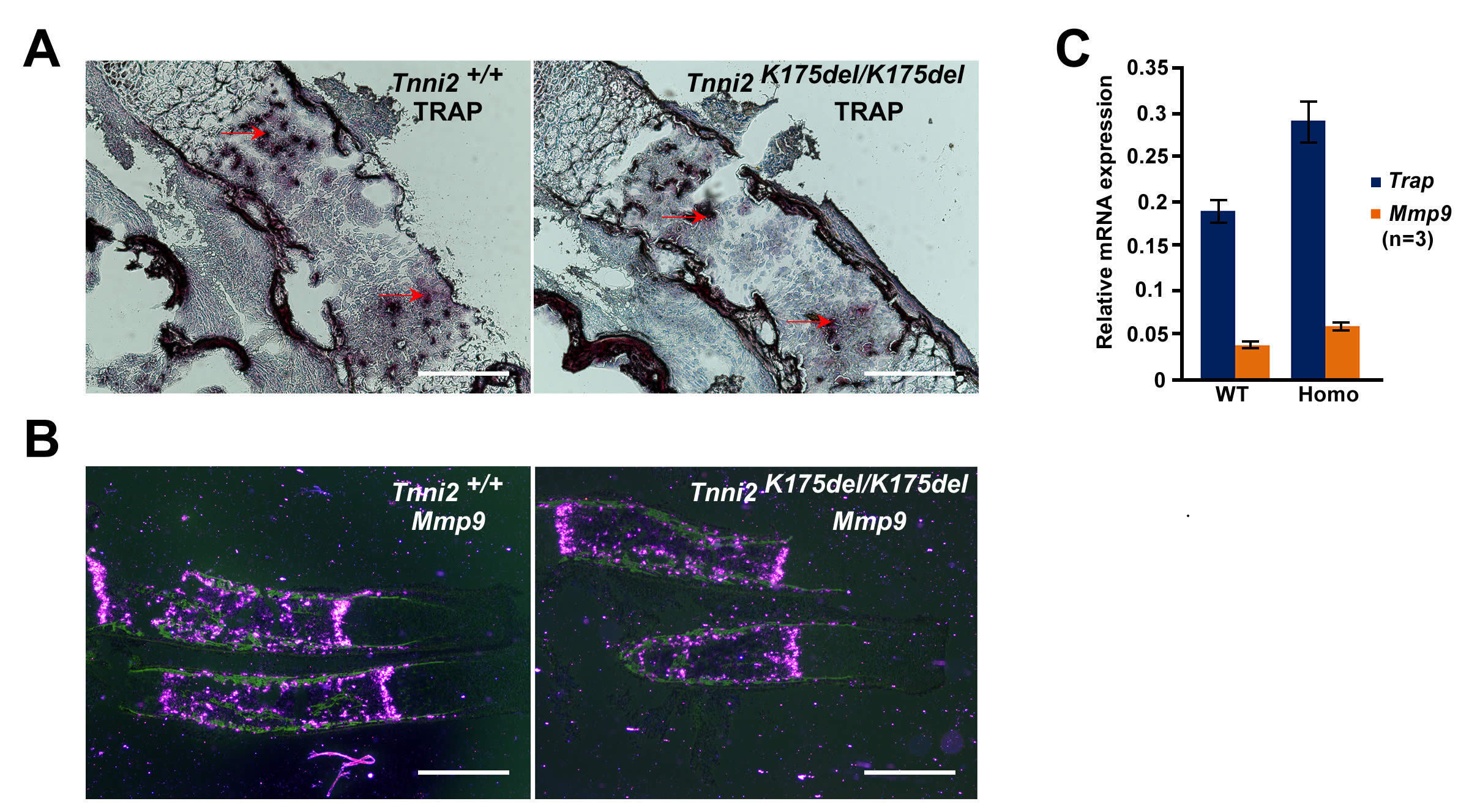

Supplement: Figure S18 — Activation of osteoclasts had no difference between homozygous mutant mice and wild-type littermates. (A) Tartrate-resistant acid phosphatase (TRAP) staining showed activated osteoclasts at E16.5 homozygous mutant radii (n = 3) and wild-type controls (n = 3). Osteoclasts were stained as violet (arrows). (B) In situ hybridization analyses with antisense Mmp9 riboprobe on histological sections of radii and ulnae from E17.5 homozygous mutant embryos (n = 2) and wild-type controls (n = 2). (C) qPCR analyses assessed the mRNA level of Mmp9 and Trap (normalized to Gapdh) in the radii and ulnae from newborn Tnni2K175del/K175del mice and wild-type littermates. mean±s.d., Scale bar, 100 µm (A, B). (JPG) [file pgen.1004589.s018.jpg]

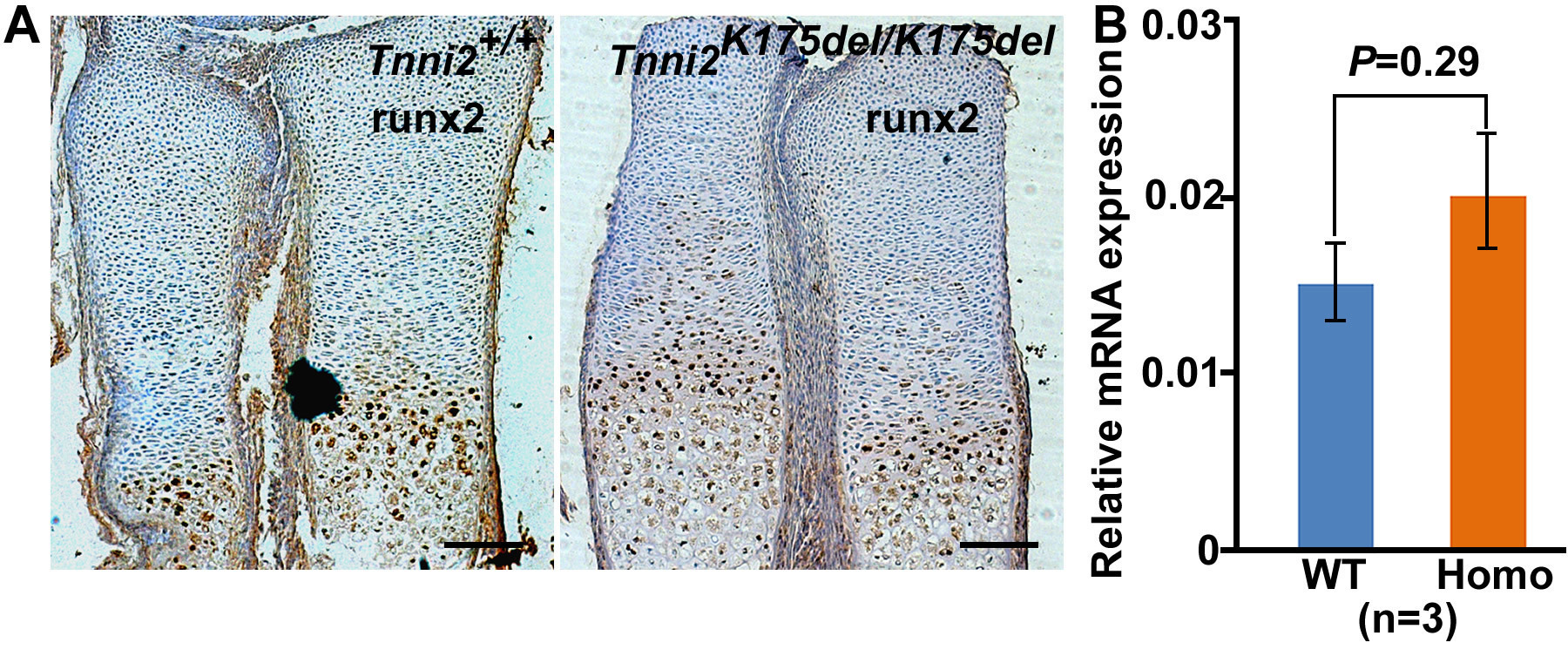

Supplement: Figure S19 — The expression level of Runx2. (A) Immunostaining analyses showed that the runx2 protein had no significant difference at hypertrophic cell zones in radii of E16.5 homozygous mutants and wild-type littermates (n = 3). (B) Microarray expression data of Runx2 in the radii and ulnae of newborn Tnni2K175del/K175del mice (n = 3) and wild-type littermates (n = 3). Student's t-test, mean±s.d., Scale bar, 100 µm (A) (JPG) [file pgen.1004589.s019.jpg]
