# Supplementary material for: A Gain-of-Function Mutation in Tnni2 Impeded Bone Development through Increasing Hif3a Expression in DA2B Mice
Source: PLoS Genet. 2014 Oct 23;10(10):e1004589. doi: 10.1371/journal.pgen.1004589 (PMC4207604; doi:10.1371/journal.pgen.1004589)
Supplement: Table S1 — The summary of significantly differential expression genes in the microarray dataset. (PDF) [file pgen.1004589.s020.pdf]

Table S1 Significantly differential gene expression in newborn radii and ulnae of *Tnni2*^{K175del} mutants versus wild type littermates (SAM analysis; Score≥ 2 or Score≤ -2; fold≥ 2)

Gene Symbol	Probe Set ID	Score(d)	Fold Change	q-value(%)
Bmf	10486112	5.261	2.118	0
8430408G22Rik	10541071	4.987	4.271	0
Msmp	10512594	4.931	3.068	0
Ddit4	10369290	3.977	4.619	16.109
Hif3a	10560329	3.789	3.324	16.109
Klhl6	10438445	3.694	2.029	16.109
Ccng2	10523297	3.640	2.095	16.109
Bmper	10583870	3.292	2.003	16.109
Gas5	10351026	3.221	2.709	16.109
Pik3r1	10411782	3.122	2.363	16.109
Pik3ip1	10373740	3.086	2.906	16.109
2610008E11Rik	10370544	3.082	2.089	16.109
Angptl7	10518526	3.036	24.086	16.109
Pcmtd2	10479726	2.957	2.442	16.109
Ogt	10601235	2.932	2.267	16.109
Itgbl1	10417212	2.922	2.271	16.109
Snrk	10590445	2.921	2.090	16.109
Adh1	10496438	2.896	4.868	16.109
Trp53inp1	10503259	2.850	4.985	16.109
Tsc22d3	10606989	2.846	4.747	16.109
---	10339823	2.822	2.367	16.109
Clk1	10354816	2.785	2.733	16.109
Id2	10399691	2.752	2.163	17.490
Jak2	10462363	2.653	2.049	18.740
Sat1	10607467	2.609	2.421	20.869
Rasl11b	10522467	2.561	2.435	20.869
Klhl24	10434302	2.545	3.016	20.869
---	10341722	2.545	2.170	20.869
Jhdm1d	10544148	2.488	2.080	20.869
Glul	10350753	2.466	3.765	20.869
Lifr	10422822	2.446	2.498	20.869
Gabarapl1	10542200	2.410	2.460	20.869
Ndrg2	10419578	2.367	2.759	20.869
Hsd17b11	10531919	2.350	2.030	20.869
Arrdc3	10406407	2.317	3.010	20.869
Mertk	10475890	2.313	2.144	20.869
Chodl	10436623	2.307	2.031	20.869
Mt1	10574027	2.269	2.862	20.869
Spock2	10363415	2.261	3.144	20.869

Glul	10374453	2.252	3.547	21.662
Zbtb16	10593225	2.234	5.254	21.662
---	10344166	2.234	2.045	21.662
Ubxn7	10435031	2.222	2.201	21.662
Pdgfra	10522503	2.214	2.180	21.662
Appl2	10371356	2.194	2.201	21.662
Net1	10407420	2.187	2.039	21.662
Sesn1	10362811	2.159	4.520	21.662
Lnpep	10448094	2.144	2.041	21.662
Abca1	10512949	2.139	3.338	21.662
Ezh1	10391378	2.136	2.168	21.662
Fkbp5	10449452	2.131	6.163	21.662
Egln3	10400304	2.126	2.511	21.662
Klf15	10540028	2.099	2.826	21.662
Ctsk	10494262	2.078	2.200	21.662
Mylip	10404874	2.077	2.035	21.662
Gab1	10579925	2.047	2.286	23.925
---	10342094	2.033	2.028	23.925
Spp1	10523717	2.005	2.455	23.925
Rbl2	10573867	2.005	2.259	23.925
---	10339129	-2.486	0.478	0
Spon2	10529264	-2.524	0.403	0
Mybpc2	10562784	-2.782	0.408	0
---	10598027	-2.875	0.342	0
---	10340242	-3.051	0.496	0
---	10338724	-3.534	0.352	0
