# Supplementary material for: A Gain-of-Function Mutation in Tnni2 Impeded Bone Development through Increasing Hif3a Expression in DA2B Mice
Source: PLoS Genet. 2014 Oct 23;10(10):e1004589. doi: 10.1371/journal.pgen.1004589 (PMC4207604; doi:10.1371/journal.pgen.1004589)
Supplement: Table S2 — Immunoprecipitation and mass spectrometry analyses revealed potential proteins interaction with tnni2 using the nuclear extracts from 293T cells ectopically expressing wild-type tnni2-GFP fusion protein. KPNB1, IPO5, IPO8, IPO9, TNPO1 and TNPO2 were highlighted in red. TNNI2 was marked in bold. (PDF) [file pgen.1004589.s021.pdf]

Table S2 The mass spectrometry analyses of the potential proteins which interact with TNNI2, using the nuclear extracts from 293T cells ectopically expressing wild-type tnni2-GFP fusion protein.

**Sample ID**	**Accession number**	**Protein description**	**Mascot score**	**MW**
**NE1**	IPI00003865	HSPA8 Isoform 1 of Heat shock cognate 71 kDa protein	6306	70854
	IPI00304925	HSPA1A;HSPA1B Heat shock 70 kDa protein 1A/1B	5865	70009
	IPI00911039	HSPA1A;HSPA1B cDNA FLJ54408, highly similar to Heat shock 70 kDa protein 1	4731	63885
	IPI00003362	HSPA5 HSPA5 protein	2294	72377
	IPI00909073	cDNA FLJ53752, highly similar to Heat shock 70 kDa protein 1	2109	45099
	IPI00007702	HSPA2 Heat shock-related 70 kDa protein 2	1862	69978
	IPI00939526	HSPA1A;HSPA1B Putative uncharacterized protein ENSP00000410381	1557	25887
	IPI00339269	HSPA6 Heat shock 70 kDa protein 6	1493	70984
	IPI00002966	HSPA4 Heat shock 70 kDa protein 4	1278	94271
	IPI00001639	KPNB1 Importin subunit beta-1	1214	97108
	IPI00793443	IPO5 Isoform 1 of Importin-5	1072	123550
	IPI00332371	PFKL Isoform 1 of 6-phosphofructokinase, liver type	1044	84964
	IPI00009790	PFKP 6-phosphofructokinase type C	827	85542
	IPI00185146	IPO9 Importin-9	785	115889
	IPI00554737	PPP2R1A Serine/threonine-protein phosphatase 2A 65 kDa regulatory subunit A alpha isoform	763	65267
	IPI00298961	XPO1 Exportin-1	724	123306
	IPI00005711	HDAC6 cDNA FLJ56474, highly similar to Histone deacetylase 6	715	132696
	IPI00784295	HSP90AA1 Isoform 1 of Heat shock protein HSP 90-alpha	702	84607
	IPI00743142	PFKM Isoform 1 of 6-phosphofructokinase, muscle type	633	85128
	IPI00295485	HSPA4L Heat shock 70 kDa protein 4L	555	94426
	IPI00290770	CCT3 chaperonin containing TCP1, subunit 3 isoform b	544	60424

IPI00216746	HNRNPK Isoform 2 of Heterogeneous nuclear ribonucleoprotein K	509	50996
IPI00168184	PPP2R1A	508	56812
IPI00293464	DDB1;LOC100290337 DNA damage-binding protein 1	491	126887
IPI00290142	CTPS CTP synthase 1	487	66648
IPI00302925	CCT8 59 kDa protein	485	59440
IPI00012268	PSMD2 26S proteasome non-ATPase regulatory subunit 2	450	100136
IPI00414676	HSP90AB1 Heat shock protein HSP 90-beta	443	83212
IPI00295851	COPB1 Coatomer subunit beta	430	107074
IPI00645452	TUBB Tubulin, beta	391	47736
IPI00384456	MSH6 Isoform GTBP-N of DNA mismatch repair protein Msh6	380	152689
IPI00794211	UBC;RPS27A;UBB 18 kDa protein	367	17566
IPI00007752	TUBB2C Tubulin beta-2C chain	360	49799
IPI00644712	XRCC6 ATP-dependent DNA helicase 2 subunit 1	349	69799
IPI00908817	TNPO1 Isoform 3 of Transportin-1	330	96838
IPI00748807	NUP160 Isoform 1 of Nuclear pore complex protein Nup160	329	162017
IPI00295857	COPA Isoform 1 of Coatomer subunit alpha	315	138258
IPI00219990	RNASEN Isoform 2 of Ribonuclease 3	302	151199
IPI00007401	IPO8 Importin-8	296	119861
IPI00479778	SMEK1 Isoform 2 of Serine/threonine-protein phosphatase 4 regulatory subunit 3A	279	93837
IPI00022744	CSE1L Isoform 1 of Exportin-2	279	110346
IPI00164672	DCP1A Putative uncharacterized protein DCP1A	273	63397
IPI00395694	TNPO3 Isoform 2 of Transportin-3	269	104136
IPI00479786	KHSRP KH-type splicing regulatory protein	267	73070
IPI00889541	DDX17 Isoform 4 of Probable ATP-dependent RNA helicase DDX17	266	80222
IPI00397904	NUP93 Nuclear pore complex protein Nup93	265	93430
IPI00290566	TCP1 T-complex protein 1 subunit alpha	261	60306

IPI00002557	COPG2 Coatomer subunit gamma-2	259	97560
IPI00790530	NUP85 Nuclear pore complex protein Nup85	255	74971
IPI00010720	CCT5 T-complex protein 1 subunit epsilon	249	59633
IPI00419856	TNPO2 transportin 2 (importin 3, karyopherin beta 2b) isoform 3	247	100343
IPI00218993	HSPH1 Isoform Beta of Heat shock protein 105 kDa	247	92057
IPI00555743	API5 Isoform 3 of Apoptosis inhibitor 5	244	41994
IPI00100160	CAND1 Isoform 1 of Cullin-associated NEDD8-dissociated protein 1	243	136289
IPI00069232	GPRASP2 G-protein coupled receptor-associated sorting protein 2	233	93715
**IPI00216236**	**TNNI2 Troponin I, fast skeletal muscle**	230	21325
IPI00289758	CAPN2 Calpain-2 catalytic subunit	227	79959
IPI00017375	SEC23A Protein transport protein Sec23A	215	86105
IPI00017617	DDX5 Probable ATP-dependent RNA helicase DDX5	213	69105
IPI00008575	KHDRBS1 Isoform 1 of KH domain-containing, RNA-binding, signal transduction-associated protein 1	204	48197
IPI00008240	MARS Methionyl-tRNA synthetase, cytoplasmic	200	101052
IPI00027834	HNRNPL Heterogeneous nuclear ribonucleoprotein L	193	64092
IPI00017303	MSH2 DNA mismatch repair protein Msh2	192	104677
IPI00909344	KHSRP cDNA FLJ51330, highly similar to Far upstream element-binding protein 2	191	38106
IPI00216348	DYNC1I2 Isoform 2C of Cytoplasmic dynein 1 intermediate chain 2	186	68384
IPI00298520	ARCN1 Coatomer subunit delta variant 2	179	61587
IPI00030774	TBCD Isoform 4 of Tubulin-specific chaperone D	178	138508
IPI00220288	MTA3 Isoform 2 of Metastasis-associated protein MTA3	176	58776
IPI00291200	NUP133 Nuclear pore complex protein Nup133	171	128898
IPI00300074	FARSB Phenylalanyl-tRNA synthetase beta chain	169	66088
IPI00186290	EEF2 Elongation factor 2	166	95277
IPI00220219	COPB2 Coatomer subunit beta~	164	102422
IPI00783982	COPG Coatomer subunit gamma	163	97655

IPI00215637	DDX3X ATP-dependent RNA helicase DDX3X	162	73198
IPI00218343	TUBA1C Tubulin alpha-1C chain	159	49863
IPI00009315	ACBD3 Golgi resident protein GCP60	152	60556
IPI00152998	LRRC40 Leucine-rich repeat-containing protein 40	149	68207
IPI00465233	EIF3L Eukaryotic translation initiation factor 3, subunit E interacting protein	148	70857
IPI00376317	EDC4 Isoform 1 of Enhancer of mRNA-decapping protein 4	144	151567
IPI00216230	TMPO Lamina-associated polypeptide 2, isoform alpha	141	75446
IPI00027626	CCT6A T-complex protein 1 subunit zeta	138	57988
IPI00305438	VPS16 Isoform 1 of Vacuolar protein sorting-associated protein 16 homolog	136	94634
IPI00220834	XRCC5 ATP-dependent DNA helicase 2 subunit 2	135	82652
IPI00007402	IPO7 Importin-7	134	119440
IPI00018465	CCT7 T-complex protein 1 subunit eta	129	59329
IPI00215764	MTA1 Isoform Short of Metastasis-associated protein MTA1	128	48986
IPI00640703	XPO5 Isoform 1 of Exportin-5	128	136222
IPI00304935	SAAL1 Similar to Protein SAAL1. Isoform 2	126	53621
IPI00054042	GTF2I Isoform 1 of General transcription factor II-I	122	112346
IPI00218342	MTHFD1 C-1-tetrahydrofolate synthase, cytoplasmic	122	101495
IPI00299904	MCM7 Isoform 1 of DNA replication licensing factor MCM7	121	81257
IPI00220644	PKM2 Isoform M1 of Pyruvate kinase isozymes M1/M2	121	58025
IPI00293616	DDX3Y ATP-dependent RNA helicase DDX3Y	120	73108
IPI00246058	PDCD6IP Programmed cell death 6-interacting protein	120	95963
IPI00301107	IPO11 Importin-11	117	112463
IPI00449049	PARP1 Poly [ADP-ribose] polymerase 1	113	113012
IPI00219352	CBS Isoform 1 of Cystathionine beta-synthase	112	60548
IPI00033516	TUBGCP3 Isoform 1 of Gamma-tubulin complex component 3	111	103506
IPI00179057	CUL4B Isoform 1 of Cullin-4B	111	102235

IPI00154451	MMS19 cDNA FLJ55586, highly similar to MMS19-like protein	109	115675
IPI00001985	VPS18 Isoform 1 of Vacuolar protein sorting-associated protein 18 homolog	108	110116
IPI00300371	SF3B3 Isoform 1 of Splicing factor 3B subunit 3	105	135492
IPI00075081	FANCD2 Isoform 1 of Fanconi anemia group D2 protein	103	166356
IPI00455599	HSP90AB2P Similar to Heat shock protein HSP 90-beta	103	49092
IPI00165261	SCFD1 Sec1 family domain-containing protein 1	102	72334
IPI00011631	ZW10 Centromere/kinetochore protein zw10 homolog	100	88773
IPI00396370	EIF3B Isoform 1 of Eukaryotic translation initiation factor 3 subunit B	100	92424
IPI00010740	SFPQ Isoform Long of Splicing factor, proline- and glutamine-rich	99	76102
IPI00100984	HEATR3 Isoform 1 of HEAT repeat-containing protein 3	99	74535
IPI00059279	EXOC4 Exocyst complex component 4	98	110429
IPI00908375	SEC23A cDNA FLJ52821, highly similar to Protein transport protein Sec23A	98	44992
IPI00018009	EDC3 Enhancer of mRNA-decapping protein 3	97	56042
IPI00658145	C7orf27 Isoform 1 of HEAT repeat-containing protein C7orf27	96	88063
IPI00028005	NUP107 Nuclear pore complex protein Nup107	95	106307
IPI00013452	EPRS Bifunctional aminoacyl-tRNA synthetase	94	170483
IPI00156374	IPO4 Isoform 1 of Importin-4	92	118640
IPI00008248	ANAPC7 anaphase-promoting complex subunit 7 isoform a	91	66813
IPI00945733	KPNA1 Putative uncharacterized protein KPNA1	89	36807
IPI00302927	CCT4;ILK-2 T-complex protein 1 subunit delta	89	57888
IPI00303292	KPNA1 Importin subunit alpha-1	88	60211
IPI00220901	TBC1D4 TBC1 domain family member 4	86	146514
IPI00219420	SMC3 Structural maintenance of chromosomes protein 3	84	141454
IPI00103654	PPP4R2 Isoform 1 of Serine/threonine-protein phosphatase 4 regulatory subunit 2	84	46869
IPI00878298	EIF3L Eukaryotic translation initiation factor 3, subunit E interacting protein	82	14717
IPI00007208	DDX41 Probable ATP-dependent RNA helicase DDX41	82	69793

IPI00328257	AP1B1 Isoform A of AP-1 complex subunit beta-1	81	104570
IPI00103483	COBRA1 Negative elongation factor B	78	65655
IPI00747053	ERCC3 TFIIH basal transcription factor complex helicase XPB subunit	77	89221
IPI00414197	WDR26 Isoform 1 of WD repeat-containing protein 26	76	72079
IPI00015195	CSTF3 Cleavage stimulation factor subunit 3	75	82869
IPI00178972	DCAF11 Isoform 1 of DDB1- and CUL4-associated factor 11	75	61631
IPI00759723	RARS Isoform Monomeric of Arginyl-tRNA synthetase, cytoplasmic	74	67098
IPI00154283	KIAA1524 Isoform 1 of Protein CIP2A	73	102121
IPI00783781	NUP205 Nuclear pore complex protein Nup205	73	227775
IPI00156791	KLHL22 Isoform 1 of Kelch-like protein 22	72	71621
IPI00639961	BAG3 Putative uncharacterized protein BAG3	72	34738
IPI00470482	EXOC7 cDNA FLJ58532, highly similar to Exocyst complex component 7	71	74626
IPI00033022	DNM2 Isoform 1 of Dynamin-2	71	98003
IPI00073179	VPS33A Vacuolar protein sorting-associated protein 33A	70	67568
IPI00299608	PSMD1 Isoform 1 of 26S proteasome non-ATPase regulatory subunit 1	70	105769
IPI00018350	MCM5 DNA replication licensing factor MCM5	69	82233
IPI00414972	PKNOX2 Homeobox protein PKNOX2	68	51995
IPI00293655	DDX1 ATP-dependent RNA helicase DDX1	66	82380
IPI00014235	RAB3GAP1 Isoform 1 of Rab3 GTPase-activating protein catalytic subunit	66	110454
IPI00070643	FAF1 Isoform Long of FAS-associated factor 1	65	73908
IPI00027230	HSP90B1 Endoplasmin	65	92411
IPI00746351	DIS3 Isoform 1 of Exosome complex exonuclease RRP44	65	108934
IPI00026625	NUP155 Isoform 1 of Nuclear pore complex protein Nup155	64	155100
IPI00028357	XPO4 Exportin-4	63	130056
IPI00607814	XPNPEP1 X-Pro aminopeptidase 1, soluble isoform 2	63	72061
IPI00012439	HDAC10 Isoform 1 of Histone deacetylase 10	61	71399

IPI00301180	SLC12A5 Isoform 2 of Solute carrier family 12 member 5	61	123431
IPI00019427	EXOC1 Isoform 1 of Exocyst complex component 1	61	101917
IPI00014311	CUL2 cDNA FLJ56037, highly similar to Cullin-2	60	89446
IPI00016910	EIF3CL;EIF3C Eukaryotic translation initiation factor 3 subunit C	58	105278
IPI00237671	NEFL Neurofilament light polypeptide	58	61479
IPI00794610	DNAJC7 DnaJ (Hsp40) homolog, subfamily C, member 7 isoform 2	58	50065
IPI00157757	MICAL1 cDNA FLJ56489	58	119747
IPI00414434	SRRT Arsenite-resistance protein ARS2	58	25498
IPI00072224	RINT1 RAD50-interacting protein 1	57	90574
IPI00031023	FLII Protein flightless-1 homolog	57	144659
IPI00294879	RANGAP1 Ran GTPase-activating protein 1	57	63502
IPI00029705	TUBGCP2 Gamma-tubulin complex component 2	57	102469
IPI00477040	NUP188 Isoform 1 of Nucleoporin NUP188 homolog	57	195917
IPI00943982	SAMHD1 Isoform 2 of SAM domain and HD domain-containing protein 1	56	69400
IPI00880053	- 60 kDa chaperonin	55	48763
IPI00166331	ANKRD35 Ankyrin repeat domain-containing protein 35	54	109898
IPI00022629	PRC1 Isoform 1 of Protein regulator of cytokinesis 1	53	71562
IPI00011603	PSMD3 26S proteasome non-ATPase regulatory subunit 3	52	60939
IPI00020127	RPA1 Replication protein A 70 kDa DNA-binding subunit	52	68095
IPI00445697	LOC100129380 Conserved hypothetical protein	51	16435
IPI00793999	TBC1D15 TBC1 domain family, member 15 isoform 2	51	78522
IPI00184533	USP11 Ubiquitin carboxyl-terminal hydrolase 11	51	109747
IPI00168691	SHCBP1 SHC SH2 domain-binding protein 1	51	75612
IPI00306290	XPOT Exportin-T	50	109893
IPI00003519	EFTUD2 116 kDa U5 small nuclear ribonucleoprotein component	50	109366
IPI00023073	XRCC3 DNA repair protein XRCC3	50	37826

IPI00008247	ANAPC5 Isoform 1 of Anaphase-promoting complex subunit 5	49	85023
IPI00007765	HSPA9 Stress-70 protein, mitochondrial	48	73635
IPI00178512	C1orf112 Isoform 1 of Uncharacterized protein C1orf112	48	96492
IPI00328298	SMC4 Isoform 2 of Structural maintenance of chromosomes protein 4	48	140191
IPI00029236	IGFBP5 Insulin-like growth factor-binding protein 5	48	30550
IPI00412224	WDR11 Bromodomain and WD repeat-containing protein 2	47	136598
IPI00005791	NDC80 Kinetochore protein NDC80 homolog	47	73867
IPI00329679	ZWILCH Isoform 1 of Protein zwilch homolog	46	67172
IPI00440122	MSH6 Sperm protein	46	36838
IPI00031982	NCKAP1 Isoform 1 of Nck-associated protein 1	46	128707
IPI00007347	COG5 Isoform 1 of Conserved oligomeric Golgi complex subunit 5	45	92661
IPI00917208	CUL3 Putative uncharacterized protein CUL3	45	14513
IPI00171903	HNRNPM Isoform 1 of Heterogeneous nuclear ribonucleoprotein M	45	77464
IPI00382458	CUL3 Isoform 2 of Cullin-3	45	86179
IPI00376322	KIAA0564 Isoform 2 of Uncharacterized protein KIAA0564	45	116867
IPI00328890	DNAJC4 DnaJ homolog subfamily C member 4	44	27576
IPI00013871	RRM1 Ribonucleoside-diphosphate reductase large subunit	44	90013
IPI00871370	CUL5 Putative uncharacterized protein CUL5 (Fragment)	44	90793
IPI00009464	EXOSC10 Isoform 1 of Exosome component 10	44	100768
IPI00647217	SKIV2L2 Superkiller viralicidic activity 2-like 2	44	117729
IPI00007927	SMC2 Isoform 1 of Structural maintenance of chromosomes protein 2	44	135572
IPI00181702	SFRS15 Isoform 1 of Splicing factor, arginine/serine-rich 15	44	125790
IPI00216999	C14orf21 Pumilio domain-containing protein C14orf21	44	69394
IPI00022891	SLC25A4 ADP/ATP translocase 1	44	33043
IPI00014220	HAUS5 Isoform 1 of HAUS augmin-like complex subunit 5	44	71638
IPI00908905	- cDNA FLJ58872, highly similar to Keratin, type I cytoskeletal 15	43	23776

IPI00337307	TRMT2A Isoform 1 of tRNA (uracil-5-)-methyltransferase homolog A (Fragment)	43	68682
IPI00164838	- 25 kDa protein	43	24799
IPI00000856	FERMT2 Isoform 1 of Fermitin family homolog 2	43	77811
IPI00029159	MRE11A cDNA FLJ38069 fis,	42	81020
IPI00165496	NGLY1 Isoform 2 of Peptide-N(4)-(N-acetyl-beta-glucosaminyl)asparagine amidase	42	72240
IPI00152318	CCDC110 Isoform 1 of Coiled-coil domain-containing protein 110	41	96665
IPI00016725	GTF3C4 General transcription factor 3C polypeptide 4	41	91923
IPI00929375	HDAC2 Isoform 1 of Histone deacetylase 2	41	55329
IPI00395476	PRMT10 Isoform 1 of Putative protein arginine N-methyltransferase 10	40	94441

Note: a group set of nucleocytoplasmic transporters including KPNB1, IPO5, IPO8, IPO9, TNPO1 and TNPO2 was marked using red color. TNNI2 was marked as boldface.
