# Supplementary material for: A Gain-of-Function Mutation in Tnni2 Impeded Bone Development through Increasing Hif3a Expression in DA2B Mice
Source: PLoS Genet. 2014 Oct 23;10(10):e1004589. doi: 10.1371/journal.pgen.1004589 (PMC4207604; doi:10.1371/journal.pgen.1004589)
Supplement: Table S3 — Primers for ChIP-PCR. (PDF) [file pgen.1004589.s022.pdf]

Table S3 The primers for ChIP-PCR to detect the Part1 fragment at *Hif3α* promoter

| No | Forward primer | Reverse primer |
|---|---|---|
| 1 | 5' GGTAGTGCCCCTGTCTCCTGCTTAG 3' | 5' GCTCTTCACTGTGATATTGAGATTC 3' |
